# Supplementary material for: Adolescent undernutrition in South Asia: a scoping review protocol
Source: BMJ Open. 2020 Jan 23;10(1):e031955. doi: 10.1136/bmjopen-2019-031955 (PMC7044844; doi:10.1136/bmjopen-2019-031955)
Supplement: Supplementary data [file bmjopen-2019-031955supp001.pdf]

Manuscript v1 Sara Estecha Querol

Appendix 1: search strategy

Medline: ovid

	Searches
1	South Asia*.mp.
2	South East Asia*.mp.
3	exp Afghanistan/
4	exp Bangladesh/
5	exp Bhutan/
6	exp India/
7	Maldives.mp.
8	exp Nepal/
9	exp Pakistan/
10	exp Sri Lanka/
11	1 or 2 or 3 or 4 or 5 or 6 or 7 or 8 or 9 or 10
12	exp Thinness/
13	exp malnutrition/ or exp deficiency diseases/
14	(undernutrition or under nutrition or under-nutrition or stunting or stunted or underweight or under-weight or under weight or thinness or micronutrient deficienc*).mp. [mp=title, abstract, original title, name of substance word, subject heading word, floating sub-heading word, keyword heading word, organism supplementary concept word, protocol supplementary concept word, rare disease supplementary concept word, unique identifier, synonyms]
15	12 or 13 or 14
16	(adolescen* or teen* or youth* or student* or juvenile* or underage or young people or young person or young adult or girl or boy).mp
17	exp Adolescent/
18	16 or 17
19	11 and 15 and 18

Embase: ovid

	Searches
1	exp South Asia/
2	exp Southeastern Asia/
3	exp Afghanistan/
4	exp Bangladesh/
5	exp Bhutan/
6	exp India/
7	exp Maldives/
8	exp Nepal/
9	exp Pakistan/
10	exp Sri Lanka/
11	1 or 2 or 3 or 4 or 5 or 6 or 7 or 8 or 9 or 10
12	exp malnutrition/
13	exp underweight/
14	exp nutritional deficiency/
15	(undernutrition or under nutrition or under-nutrition or stunting or stunted or underweight or under-weight or under weight or thinness or micronutrient deficienc*).mp. [mp=title, abstract, heading word, drug trade name, original title, device manufacturer, drug manufacturer, device trade name, keyword, floating subheading word, candidate term word]
16	12 or 13 or 14 or 15
17	(adolescen* or teen* or youth* or student* or juvenile* or underage or young people or young person or young adult or girl or boy).mp
18	exp adolescence/
19	17 or 18
20	11 and 16 and 19

Manuscript v1 Sara Estecha Querol

Cochrane Library

	Searches
1.	South Asia or Afghanistan or Bangladesh or Bhutan or India or Maldives or Nepal or Pakistan or Sri Lanka
2.	malnutrition or undernutrition or under nutrition or under-nutrition or stunting or stunted or underweight or under-weight or under weight or thinness or micronutrient deficienc*
3.	adolescen* or teen* or youth* or student* or juvenile* or underage or young people or young person or young adult or girl or boy
4	#1 AND #2 AND #3

Web of Science

	Searches
1.	TS=(South Asia or Afghanistan or Bangladesh or Bhutan or India or Maldives or Nepal or Pakistan or Sri Lanka)
2.	TS=(malnutrition or undernutrition or under nutrition or under-nutrition or stunting or stunted or underweight or under-weight or under weight or thinness or micronutrient deficienc*)
3.	TS=(adolescen* or teen* or youth* or student* or juvenile* or underage or young people or young person or young adult or girl or boy)
4.	#3 AND #2 AND #1

CINAHL

	Searches
1	“South Asia”
2	(MH “Asia, Western+”)
3	“Afghanistan”
4	“Bangladesh”
5	“Bhutan”
6	“India”
7	“Maldives”
8	“Nepal”
9	“Pakistan”
10	“Sri Lanka”
11	S1 OR S2 OR S3 OR S4 OR S5 OR S6 OR S7 OR S8 OR S9 OR S10
12	(MH "Malnutrition") OR "malnutrition"
13	(MH "Thinness") OR "thinness"
14	"micronutrient deficiency" OR (MH "Vitamin B6 Deficiency") OR (MH "Vitamin B12 Deficiency") OR (MH "Folic Acid Deficiency") OR (MH "Ascorbic Acid Deficiency") OR (MH "Vitamin B Deficiency") OR (MH "Riboflavin Deficiency") OR (MH "Vitamin E Deficiency") OR (MH "Thiamine Deficiency") OR (MH "Vitamin D Deficiency") OR (MH "Vitamin A Deficiency")
15	"undernutrition"
16	"stunting"
17	S11 OR S12 OR S13
18	"adolescents or teenagers or young adults" OR (MH "Young Adult") OR (MH "Adolescence")
	S10 AND S14 AND S15

Manuscript v1 Sara Estecha Querol

Psychinfo

	Searches
1	South Asia*.mp.
2	Afghanistan.mp.
3	Bangladesh.mp.
4	Bhutan.mp.
5	India.mp.
6	Maldives.mp.
7	Nepal.mp.
8	Pakistan.mp.
9	Sri Lanka.mp.
10	1 or 2 or 3 or 4 or 5 or 6 or 7 or 8 or 9
11	exp Nutritional Deficiencies/
12	(malnutrition or undernutrition or under nutrition or under-nutrition or stunting or stunted or underweight or under-weight or under weight or thinness or micronutrient deficienc*).mp. [mp=title, abstract, heading word, table of contents, key concepts, original title, tests & measures]
13	11 or 12
14	(adolescen* or teen* or youth* or student* or juvenile* or underage or young people or young person or young adult or girl or boy).mp
15	10 and 13 and 14

Scopus

((TITLE-ABS-KEY (afghanistan) OR TITLE-ABS-KEY (bangladesh) OR TITLE-ABS-KEY (bhutan) OR TITLE-ABS-KEY (india) OR TITLE-ABS-KEY (maldives) OR TITLE-ABS-KEY (nepal) OR TITLE-ABS-KEY (pakistan) OR TITLE-ABS-KEY (sri AND lanka) OR TITLE-ABS-KEY (south AND asia*))) AND ((TITLE-ABS-KEY (youth*) OR TITLE-ABS-KEY (adolescen*) OR TITLE-ABS-KEY (student*) OR TITLE-ABS-KEY (juvenile*) OR TITLE-ABS-KEY (underage) OR TITLE-ABS-KEY (young AND people) OR TITLE-ABS-KEY (young AND person) OR TITLE-ABS-KEY (young AND adult) OR TITLE-ABS-KEY (girl) OR TITLE-ABS-KEY (boy) OR TITLE-ABS-KEY (teen*))) AND ((TITLE-ABS-KEY (malnutrition) OR TITLE-ABS-KEY (undernutrition) OR TITLE-ABS-KEY (under AND nutrition) OR TITLE-ABS-KEY (under-nutrition) OR TITLE-ABS-KEY (stunting) OR TITLE-ABS-KEY (stunted) OR TITLE-ABS-KEY (underweight) OR TITLE-ABS-KEY (under-weight) OR TITLE-ABS-KEY (under AND weight) OR TITLE-ABS-KEY (thinness) OR TITLE-ABS-KEY (micronutrient AND deficienc*))) AND (EXCLUDE (SUBJAREA , "BIOC") OR EXCLUDE (SUBJAREA , "ENVI") OR EXCLUDE (SUBJAREA , "IMMU") OR EXCLUDE (SUBJAREA , "PHAR") OR EXCLUDE (SUBJAREA , "ARTS") OR EXCLUDE (SUBJAREA , "ECON") OR EXCLUDE (SUBJAREA , "PSYC") OR EXCLUDE (SUBJAREA , "ENGI") OR EXCLUDE (SUBJAREA , "NEUR") OR EXCLUDE (SUBJAREA , "DENT") OR EXCLUDE (SUBJAREA , "EART") OR EXCLUDE (SUBJAREA , "CHEM") OR EXCLUDE (SUBJAREA , "ENER") OR EXCLUDE (SUBJAREA , "CENG") OR EXCLUDE (SUBJAREA , "MATH") OR EXCLUDE (SUBJAREA , "VETE") OR EXCLUDE (SUBJAREA , "BUSI") OR EXCLUDE (SUBJAREA , "DECI") OR EXCLUDE (SUBJAREA , "COMP") OR EXCLUDE (SUBJAREA , "MATE") OR EXCLUDE (SUBJAREA , "PHYS") OR EXCLUDE (SUBJAREA , "Undefined"))

Manuscript v1 Sara Estecha Querol

Grey literature sources

WHOLIS – the WHO Library Information System	http://kohahq.searo.who.int/
eLENA e-Library of Evidence for Nutrition Actions	https://www.who.int/elena/en/
Opengrey	http://www.opengrey.eu/
WHO	https://www.who.int/en
UNICEF	https://www.unicef.org/
DHS program	https://dhsprogram.com/
Planning and Development Department AJ&K	https://pndajk.gov.pk/
GHDx	http://ghdx.healthdata.org/geography/pakistan
WFP	https://www1.wfp.org/
World Bank eLibrary	https://elibrary.worldbank.org/
