# Supplementary material for: Adolescent undernutrition in South Asia: a scoping review protocol
Source: BMJ Open. 2020 Jan 23;10(1):e031955. doi: 10.1136/bmjopen-2019-031955 (PMC7044844; doi:10.1136/bmjopen-2019-031955)
Supplement: Author's manuscript [file bmjopen-2019-031955.draft_revisions.pdf]

BMJ Open

BMJ Open is committed to open peer review. As part of this commitment we make the peer review history of every article we publish publicly available.

When an article is published we post the peer reviewers' comments and the authors' responses online. We also post the versions of the paper that were used during peer review. These are the versions that the peer review comments apply to.

The versions of the paper that follow are the versions that were submitted during the peer review process. They are not the versions of record or the final published versions. They should not be cited or distributed as the published version of this manuscript.

BMJ Open is an open access journal and the full, final, typeset and author-corrected version of record of the manuscript is available on our site with no access controls, subscription charges or pay-per-view fees (http://bmjopen.bmj.com).

If you have any questions on BMJ Open's open peer review process please email info.bmjopen@bmj.com

BMJ Open

Adolescent undernutrition in South Asia: a scoping review protocol

Journal:	*BMJ Open*
Manuscript ID	bmjopen-2019-031955
Article Type:	Protocol
Date Submitted by the Author:	27-May-2019
Complete List of Authors:	Estecha Querol, Sara; University of Warwick Warwick Medical School, Academic Unit of Primary Care; University of Warwick Warwick Medical School, NIHR Global Health Research Unit in Improving Health in Slums<br>Al-Khudairy, Lena; University of Warwick Warwick Medical School, Division of Health Sciences<br>Iqbal, Romaina; Aga Khan University, Department of Community Health Sciences and Medicine<br>Johnson, Samantha ; University of Warwick Warwick Medical School,<br>Gill, Paramjit ; University of Warwick Warwick Medical School, Academic Unit of Primary Care; University of Warwick Warwick Medical School, NIHR Global Health Research Unit in Improving Health in Slums
Keywords:	South Asia, Adolescents, undernutrition, Scoping review

SCHOLARONE™
Manuscripts

BMJ

I, the Submitting Author has the right to grant and does grant on behalf of all authors of the Work (as defined in the below author licence), an exclusive licence and/or a non-exclusive licence for contributions from authors who are: i) UK Crown employees; ii) where BMJ has agreed a CC-BY licence shall apply, and/or iii) in accordance with the terms applicable for US Federal Government officers or employees acting as part of their official duties; on a worldwide, perpetual, irrevocable, royalty-free basis to BMJ Publishing Group Ltd ("BMJ") its licensees and where the relevant Journal is co-owned by BMJ to the co-owners of the Journal, to publish the Work in this journal and any other BMJ products and to exploit all rights, as set out in our licence.

The Submitting Author accepts and understands that any supply made under these terms is made by BMJ to the Submitting Author unless you are acting as an employee on behalf of your employer or a postgraduate student of an affiliated institution which is paying any applicable article publishing charge ("APC") for Open Access articles. Where the Submitting Author wishes to make the Work available on an Open Access basis (and intends to pay the relevant APC), the terms of reuse of such Open Access shall be governed by a Creative Commons licence – details of these licences and which Creative Commons licence will apply to this Work are set out in our licence referred to above.

Other than as permitted in any relevant BMJ Author's Self Archiving Policies, I confirm this Work has not been accepted for publication elsewhere, is not being considered for publication elsewhere and does not duplicate material already published. I confirm all authors consent to publication of this Work and authorise the granting of this licence.

For peer review only

TITLE PAGE

Title of the article:

Adolescent undernutrition in South Asia: a scoping review protocol

Full name, postal address, e-mail and telephone number of the corresponding author:

Sara Estecha Querol

Warwick Medical School, University of Warwick

Gibbet Hill Road, Coventry (UK) CV4 7AL

Sara.Estecha-Querol@warwick.ac.uk

+44 2476575343

Full name, department, institution, city and country of all co-authors:

Sara Estecha Querol[1,2], Lena Al-Khudairy[3], Romaina Iqbal[4], Samantha Johnson[5], Paramjit Gill[1,2].

[1] Academic Unit of Primary Care, University of Warwick, Coventry, UK

[2] NIHR Global Health Research Unit in Improving Health in Slums, University of Warwick, Coventry, UK

[3] Division of Health Sciences, Warwick Medical School, University of Warwick, Coventry, UK

[4] Department of Community Health Sciences, Aga Khan University, Karachi, Pakistan.

[5] The Library, University of Warwick, Coventry, UK

Word count (excluding title page, abstract, references, figures and tables):

2,189 words

For peer review only - http://bmjopen.bmj.com/site/about/guidelines.xhtml

TITLE

Adolescent undernutrition in South Asia: a scoping review protocol

ABSTRACT

Introduction:
Malnutrition is considered a highly prevalent issue affecting all countries around the world. While research has mainly focused on children under 5 years of age and women of reproductive age, adolescent malnutrition is not well monitored. This paper presents a scoping review protocol on adolescent malnutrition in South Asia.

Methods and analysis:
The proposed scoping review will be guided by Arksey and O'Malley's framework and The Joanna Briggs Institute Reviewers' Manual. The scoping review question, eligibility criteria and search strategy will be based on the Population, Concept and Context (PCC) strategy. We will conduct the search in electronic bibliographic databases (Medline (OVID), Embase, Cochrane Library, Web of Science, CINAHL, PsycInfo, Scopus) as well as various grey literature sources in order to synthesise and present the findings with descriptive statistics and a narrative description of both quantitative and qualitative evidence.

Dissemination and ethics:
This protocol will accurately describe the proposed scoping review that will map the evidence on adolescent malnutrition in South Asia. The proposed review aims to gather published and unpublished literature to inform policy and healthcare organizations as well as identify future research priorities in South Asia. This study protocol does not require ethical approval.

KEYWORDS: South Asia, adolescents, undernutrition, scoping review.

ARTICLE SUMMARY

Strengths and limitations of this study

- This protocol will clearly describe the proposed methods to collect and synthesise evidence on adolescent malnutrition in South Asia, and identify knowledge gaps.
- The proposed search strategy will be conducted in seven electronic databases. Grey literature sources will be also included in the proposed scoping review, such as government reports and organisation websites.

For peer review only - http://bmjopen.bmj.com/site/about/guidelines.xhtml

- This proposed scoping review describes the mapping process of all types of literature available, however quality assessment is not required in scoping reviews.

For peer review only

For peer review only - http://bmjopen.bmj.com/site/about/guidelines.xhtml

INTRODUCTION

The most recent Global Nutrition Report highlights the global burden of malnutrition and that it remains unacceptably high affecting every country in the world.(1) The World Health Assembly formulated six global nutrition targets aiming to tackle the burden of malnutrition worldwide by 2025.(2) These targets plan to decrease stunting and wasting in children, reduce anaemia in women, decrease the rates of low birth weight, increase the rates of exclusive breastfeeding in the first 6 months, and ensure that there is no increase in childhood overweight. Malnutrition refers to deficiencies, excesses or imbalances in a person's intake of energy and/or nutrients.(3) Focusing on early stages in life, the term malnutrition covers several conditions affecting child growth patterns such as stunting, wasting, underweight, micronutrient deficiencies and overweight/obesity. Stunting, or low height for age, is caused among other factors, by long-term insufficient nutrient intake, frequent infections and diseases. Wasting, or low weight for height, also indicates malnutrition prevalence among children under 5 years of age. While wasting is the result of acute significant food shortage and/or disease, stunting represents chronic malnutrition and the effects are largely irreversible. Underweight, or low weight for age, includes children under 5 with low weight for height (wasting) and low height for age (stunting) and considered a proxy indicator for undernutrition if data on wasting are not available. Micronutrient deficiencies or insufficiencies, also called "hidden hunger", indicate a lack of important vitamins and minerals. Stunting, wasting, underweight and micronutrient deficiencies constitute the term "undernutrition".(3) Overweight/obesity is another form of malnutrition that generally involves the development of non-communicable diseases. Overweight/obesity are attributed to the term "overnutrition".(3) However, grown reference data and indicators are different between children and adolescents. The World Health Organization (WHO) recommends the use of BMI for age (thinness and overweight) and height for age (stunting) to explore adolescent growth patterns.(4, 5) Stunting is defined as height-for-age <2 SDs below the WHO Child Growth Reference median. Overweight is defined as BMI-for-age >1 SD above the WHO Growth Reference median, and obesity is defined as >2 SDs above the WHO Growth Reference median. Thinness is defined as BMI-for-age <2 SDs below the WHO Growth Reference median.(5) The term thinness and underweight have been used interchangeably when making reference to adolescents' low BMI-for-age.(6, 7)

The *State of food security and nutrition in the world* reviewed the global prevalence of thinness among children aged 5–9 years and adolescents aged 10–19 years.(8) Thinness (BMI for age below 2 SD) in these age groups was associated with a higher risk of infectious diseases, delayed maturation, reduced muscular strength, work capacity and bone density later in life. In addition, thinness in adolescent girls

For peer review only - http://bmjopen.bmj.com/site/about/guidelines.xhtml

was associated with adverse pregnancy outcomes and intrauterine growth retardation. Globally, 10% of children aged 5–19 have a BMI-for-age below 2 SD. There are significant differences in the prevalence of thinness among children aged 5–19 years by region of the world. For instance, the prevalence is high at >15% in South Asian countries such as India, Afghanistan, Bangladesh, Bhutan, Nepal, Pakistan and Sri Lanka in comparison to <2% in Latin America and the Caribbean, Northern America, Europe and Oceania. The global prevalence of thinness remained fairly steady over the past decade.(8) The prevalence of stunting (height for age below 2 SD), thinness (BMI for age below 2 SD) and overweight (BMI for age above 1 SD) were globally estimated using data from the Global School-Based Student Health and Health Behavior in School-Aged Children surveys conducted in 57 low and middle income countries between 2003 and 2013, involving 129,276 adolescents aged 12 to 15 years.(9) Globally, the prevalence of stunting was 10.2%, thinness was 5.5% and overweight 21.4%. Particularly in Pakistan, the prevalence of stunting was 7.1%, thinness was 11.2% and overweight 6.4% in 2009. In India in 2007, the prevalence of stunting was 14.6%, thinness was 15.9% and overweight 11.1%. In Sri Lanka in 2008, the prevalence of stunting was 25.6%, thinness was 31.5% and overweight 4.8%.

There are around of 1.8 billion adolescents worldwide and the majority are clustered in low and middle income countries.(6) However, there is a gap in adolescent health data.(10) Whilst the large global health and nutrition surveys mainly collected household data from adolescent girls aged ≥ 15 years; the 10 -14 year old age group, unmarried adolescents, unschooled adolescents, and male adolescents have not been included in global health surveys.(10) Christian and Smith(6) highlighted the public health need to investigate adolescent nutrition deficiencies, growth and development.

Child and adolescent malnutrition in South Asia was previously reviewed.(11-14) (see a summary of reviews in Table 1). Some literature reviews(11, 13, 14) were focused on children, while Mak's systematic review(12) provided evidence on both children and adolescents. However, Mak's systematic review exclusively focused on studies examining the prevalence of underweight status in the last two decades. This scoping review protocol will include a larger pool of evidence by proposing a broader search strategy including micronutrient deficiencies as well as including grey literature without restrictions on date of publication.

	Akhtar 2016(11)	Mak 2012(12)	Pasricha 2010(13)	Khan 2007(14)	The proposed scoping review
Type of study	Literature review	Systematic review	Literature review	Literature review	Scoping review

For peer review only - http://bmjopen.bmj.com/site/about/guidelines.xhtml

Date of publication the studies included	Restrictions on date of publication not specified	1990 - 2010	Restrictions on date of publication not specified	Restrictions on date of publication not specified	Up to 2019
Context of the population	South Asia (India, Pakistan, Bangladesh, Sri Lanka and Nepal)	East Asia (China, Hong Kong, Japan, South Korea, and Taiwan), South Asia (Bangladesh, India, Indonesia, Malaysia, Nepal, Pakistan, Singapore, Thailand, and Vietnam), and West Asia (Bahrain and Iran).	South and South-East Asia (no countries are listed)	South Asia (no countries are listed)	South Asia (Afghanistan, Bangladesh, Bhutan, India, Maldives, Nepal, Pakistan, and Sri Lanka)
Age of population	Children under 5 years and school children (up to 12 years)	Children and adolescents	Children under 5 years	Children under 5 years	Adolescents 10 – 19 years
Concept of malnutrition	Undernutrition	Underweight	Undernutrition	Undernutrition	Undernutrition

Table 1: Reviews on child and adolescent malnutrition in South Asia.

REVIEW AIMS

The aim of this protocol is to describe the methodology of an up to date scoping review of the literature on adolescent malnutrition in South Asia and identifying gaps in knowledge. The proposed scoping review work could potentially be valuable to inform policy makers and healthcare organizations delivering adolescent health and nutrition actions in South Asia. It will constitute the first step in a larger research project aimed at improving adolescent health in Pakistan; these findings will inform the next quantitative and qualitative phases.

For peer review only

For peer review only - http://bmjopen.bmj.com/site/about/guidelines.xhtml

METHODS AND ANALYSIS

All study types will be included to ensure the inclusion of the majority of relevant literature.(15) The design of the proposed scoping review methodology was informed by Arksey and O'Malley's framework(15), The Joanna Briggs Institute Reviewers' Manual(16) as well as other scoping review publications.(17, 18) Arksey and O'Malley's methodological framework includes five stages to conduct a scoping review.

Stage 1: identifying the research question

We will use the Population, Concept and Context (PCC) strategy to define the title, scoping review objective, scoping review question, and inclusion criteria.(16) Therefore, our main research question will address: adolescent undernutrition in South Asia, what do we know from the existing literature?

Stage 2: identifying relevant studies

At this stage, the reviewing team (SEQ, SJ, RI, LAK, PG) discussed the key words constituting the search strategy and the criteria for inclusion and exclusion of the studies in accordance with the PCC strategy.

Databases and search strategy

An experienced research librarian (SJ) and one reviewer (SEQ) developed the search strategy by testing the keywords, MESH terms and databases to search. Finally, the reviewing team discussed and determined the final search strategy. The search will be conducted using the following electronic bibliographic databases and grey literature sources: Medline (OVID), Embase, Cochrane, Web of Science, CINAHL, PsycInfo, Scopus, the WHO Library Information System (WHOLIS), eLENA e-Library of Evidence for Nutrition Actions, and Opengrey. The websites of agencies, academic institutions and technical bodies will be also searched: World Health Organisation (WHO), United Nations International Children's Emergency Fund (UNICEF), Demographic and Health Surveys (DHS), Program, Planning and Development Department AJ&K, Global Health Data Exchange (GHDx), World Food Program (WFP), and World Bank eLibrary. In addition, the topic field expert (RI) will be consulted to identify additional relevant grey literature sources. One reviewer (SEQ) will search additional resources by hand searching the references of included studies to identify further relevant evidence. This search strategy includes South Asia AND adolescents AND undernutrition. Related terms to undernutrition were also considered in the search strategy such as stunting, thinness, underweight, and micronutrient deficiency. The search strategy was tailored to the specific requirements of each database. No restrictions on language or date of publication will be made. See full search strategy in appendix 1.

For peer review only - http://bmjopen.bmj.com/site/about/guidelines.xhtml

Eligibility criteria

Despite the broadness of the proposed scoping review research question, the eligibly criteria should be clearly defined in order to thoroughly guide the reviewers' decisions. The inclusion criteria listed below are based upon the PCC strategy:

- Population: adolescents mean age 10 – 19 years old.(19)
- Context: South Asia. The World Bank limits the South Asia region to Afghanistan, Bangladesh, Bhutan, India, Maldives, Nepal, Pakistan, and Sri Lanka.(20)
- Concept: the term malnutrition includes both under and over nutrition. However, in this proposed scoping review, malnutrition will refer only to undernutrition indicators used in adolescents (stunting, thinness or underweight, and micronutrient deficiencies(3)). Adolescent malnutrition will be assessed quantitatively using the following WHO indicators: 1) thinness or underweight (low BMI-for-age); 2) stunting (low height-for-age); and 3) micronutrient deficiencies. All studies will be included regardless of the growth references or cut-off values for micronutrients deficiency followed.
- Adolescent malnutrition in South Asia will be assessed also qualitatively, therefore qualitative studies exploring perspectives, experiences or opinions around adolescent undernutrition will be included.
- Type of studies: quantitative and qualitative studies as well as grey literature, e.g. primary research studies, reviews, government reports and guidelines.

Studies will be excluded if they include:

- Only overnutrition indicators (obesity and overweight).
- Pregnant or breastfeeding adolescents.
- Adolescent athletes.
- Adolescents with long term conditions such as diabetes, tuberculosis or HIV.
- Hospitalised adolescents.
- Intervention studies targeting treatment of a specific illness or condition such as diarrhoea.

Stage 3: study selection

The screening process of this proposed scoping review will comprise two phases. First, titles and abstract will be reviewed by two independent reviewers following a broad inclusion criteria, that is, studies looking at adolescent undernutrition in South Asia. Papers identified by either or both reviewers will be included in the next phase, which is the full-text screening. The same reviewers will

For peer review only

screen full-text studies using the eligibility criteria mentioned above. Disagreements will be resolved by either discussion or referral to a third reviewer. PRISMA flow diagram (see figure 1) will be presented to reflect the search process.

Figure 1: PRISMA Flow Diagram for scoping review process. Retrieved from Peters M, Godfrey C, McInerney P, Soares C, Khalil H, Parker D (2015) The Joanna Briggs Institute reviewers' manual 2015: methodology for JBI scoping reviews.(16)

Stage 4: charting the data

Key characteristics to be extracted from the included studies will be extracted using a pre-defined data extraction sheet. At this stage, one reviewer (SEQ) will conduct data extraction consisting of a summary table recording the key information of the selected studies.

- Author(s) / Organization
- Year of publication
- Country
- Province and city (where the study was published or conducted)
- Study design
- Aims
- Target population (age, sex)
- Sample size
- Setting
- Outcome quantitative measures: stunting, thinness or underweight, and micronutrient deficiencies. Also, note the growth references or the cut-off values for micronutrients deficiency.
- Qualitative outcomes: experiences, opinions and perspectives.
- Summary of findings.
- Recommendations of the author(s)

Stage 5: collating, summarising and reporting the results

The results of the review will be reported as a map of the data extracted in a tabular form showing descriptive statistics. The tables may display the results as distribution of studies per year, geographical area where the study was conducted, location (community, hospital or school based), target population, outcomes, and methods. In addition, a narrative description of both quantitative and qualitative results will accompany the descriptive presentation of the results by grouping the data

For peer review only - http://bmjopen.bmj.com/site/about/guidelines.xhtml

into meaningful summaries for better exposition of the findings. For the qualitative studies, thematic analysis will be used to analyse the findings if data permits. Data will be exported into Nvivo and an inductive coding approach will be followed.

DISSEMINATION AND ETHICS

This protocol will provides a high quality overview of the broad literature available on the adolescent malnutrition in South Asia. Thus, the proposed scoping review will methodologically map the evidence on this topic to identify research priorities in South Asia. The proposed review will also facilitate the identification of uncovered disparities among adolescents living in South Asia. The potential gaps could help policy makers and healthcare organizations to design new research questions as well as interventions to improve adolescent malnutrition in South Asia.

Since a scoping review involves a methodically integration and presentation of available resources, this study does not require ethics approval.

AUTHOR CONTRIBUTIONS

SEQ conceived the idea, developed the methods and wrote the first draft of the manuscript. PG, LAK and RI supervised the protocol, contributed to methods, and supported the drafting and editing the manuscript. SJ contributed meaningfully to design the search strategy (appendix 1). All authors revised and approved the final manuscript.

FUNDING STATEMENT

This work was supported by NIHR grant number 16/136/87. The research was commissioned by the National Institute of Health Research using Official Development Assistance (ODA) funding. The views expressed are those of the author(s) and not necessarily those of the NHS, the NIHR or the Department of Health and Social Care.

COMPLETING INTERESTS

None declared.

For peer review only - http://bmjopen.bmj.com/site/about/guidelines.xhtml

REFERENCES

1. Independent Expert Group of the Global Nutrition Report. 2018 Global Nutrition Report. 2018.
2. WHO. Global nutrition targets 2025: policy brief series (WHO/NMH/NHD/14.2). Geneva; 2014.
3. WHO. What is malnutrition? 2016 [Available from: http://www.who.int/features/qa/malnutrition/en/.
4. WHO. Growth reference data for 5-19 years 2007 [Available from: https://www.who.int/growthref/en/.
5. de Onis M, Onyango AW, Borghi E, Siyam A, Nishida C, Siekmann J. Development of a WHO growth reference for school-aged children and adolescents. Bulletin of the World Health Organization. 2007;85(9):660-7.
6. Christian P, Smith ER. Adolescent Undernutrition: Global Burden, Physiology, and Nutritional Risks. Annals of nutrition & metabolism. 2018;72(4):316-28.
7. NCD Risk Factor Collaboration. Worldwide trends in body-mass index, underweight, overweight, and obesity from 1975 to 2016: a pooled analysis of 2416 population-based measurement studies in 128.9 million children, adolescents, and adults. Lancet (London, England). 2017;390(10113):2627-42.
8. FAO, IFAD, UNICEF, WFP, WHO. The State of Food Security and Nutrition in the World 2018. Building climate resilience for food security and nutrition. Rome; 2018.
9. Caleyachetty R, Thomas GN, Kengne AP, Echouffo-Tcheugui JB, Schilsky S, Khodabocus J, et al. The double burden of malnutrition among adolescents: analysis of data from the Global School-Based Student Health and Health Behavior in School-Aged Children surveys in 57 low- and middle-income countries. American Journal of Clinical Nutrition. 2018;108(2):414-24.
10. Mokdad AH, Forouzanfar MH, Daoud F, Mokdad AA, El Bcheraoui C, Moradi-Lakeh M, et al. Global burden of diseases, injuries, and risk factors for young people's health during 1990-2013: a systematic analysis for the Global Burden of Disease Study 2013. Lancet (London, England). 2016;387(10036):2383-401.
11. Akhtar S. Malnutrition in South Asia-A Critical Reappraisal. Critical reviews in food science and nutrition. 2016;56(14):2320-30.
12. Mak KK, Tan SH. Underweight problems in Asian children and adolescents. European journal of pediatrics. 2012;171(5):779-85.
13. Pasricha S-R, Biggs B-A. Undernutrition among children in South and South-East Asia. 2010;46(9):497-503.
14. Khan AA, Bano N, Salam A. Child Malnutrition in South Asia:A Comparative Perspective. 2007;14(1):129-45.
15. Arksey H, O'Malley L. Scoping studies: towards a methodological framework. International Journal of Social Research Methodology. 2005;8(1):19-32.
16. Peters M, Godfrey C, McInerney P, Soares C, Khalil H, Parker D. The Joanna Briggs Institute reviewers' manual 2015: methodology for JBI scoping reviews. 2015.
17. Goudet S, Griffiths P, Bogin B, Madise N. Interventions to tackle malnutrition and its risk factors in children living in slums: a scoping review. Annals of Human Biology. 2017;44(1):1-10.
18. Rahman S, Islam MT, Alam DSJBPH. Obesity and overweight in Bangladeshi children and adolescents: a scoping review. 2014;14(1):70.
19. WHO. Adolescent health and development 2019 [Available from: http://www.searo.who.int/entity/child_adolescent/topics/adolescent_health/en/.
20. The World Bank. The World Bank in South Asia 2019 [Available from: http://www.worldbank.org/en/region/sar/overview.

For peer review only - http://bmjopen.bmj.com/site/about/guidelines.xhtml

For peer review only

For peer review only - http://bmjopen.bmj.com/site/about/guidelines.xhtml

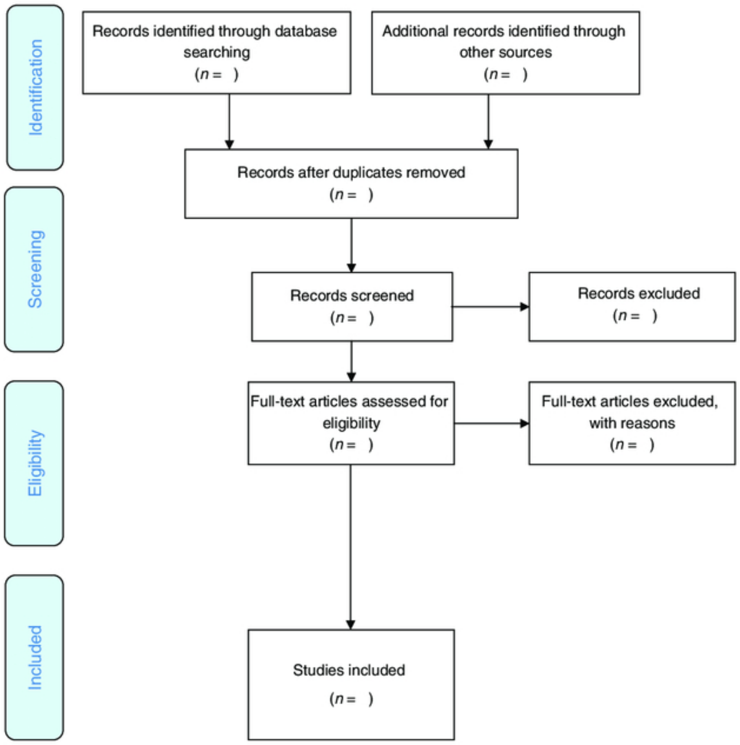

Figure 1: PRISMA Flow Diagram for scoping review process. Retrieved from Peters M, Godfrey C, McInerney P, Soares C, Khalil H, Parker D (2015) The Joanna Briggs Institute reviewers' manual 2015: methodology for JBI scoping reviews.(16)

299x251mm (72 x 72 DPI)

Appendix 1: search strategy

Medline: ovid

	Searches
1	South Asia*.mp.
2	South East Asia*.mp.
3	exp Afghanistan/
4	exp Bangladesh/
5	exp Bhutan/
6	exp India/
7	Maldives.mp.
8	exp Nepal/
9	exp Pakistan/
10	exp Sri Lanka/
11	1 or 2 or 3 or 4 or 5 or 6 or 7 or 8 or 9 or 10
12	exp Thinness/
13	exp malnutrition/ or exp deficiency diseases/
14	(undernutrition or under nutrition or under-nutrition or stunting or stunted or underweight or under-weight or under weight or thinness or micronutrient deficienc*).mp. [mp=title, abstract, original title, name of substance word, subject heading word, floating sub-heading word, keyword heading word, organism supplementary concept word, protocol supplementary concept word, rare disease supplementary concept word, unique identifier, synonyms]
15	12 or 13 or 14
16	(adolescen* or teen* or youth* or student* or juvenile* or underage or young people or young person or young adult or girl or boy).mp
17	exp Adolescent/
18	16 or 17
19	11 and 15 and 18

Embase: ovid

	Searches
1	exp South Asia/
2	exp Southeastern Asia/
3	exp Afghanistan/
4	exp Bangladesh/
5	exp Bhutan/
6	exp India/
7	exp Maldives/
8	exp Nepal/
9	exp Pakistan/
10	exp Sri Lanka/
11	1 or 2 or 3 or 4 or 5 or 6 or 7 or 8 or 9 or 10
12	exp malnutrition/
13	exp underweight/
14	exp nutritional deficiency/
15	(undernutrition or under nutrition or under-nutrition or stunting or stunted or underweight or under-weight or under weight or thinness or micronutrient deficienc*).mp. [mp=title, abstract, heading word, drug trade name, original title, device manufacturer, drug manufacturer, device trade name, keyword, floating subheading word, candidate term word]
16	12 or 13 or 14 or 15
17	(adolescen* or teen* or youth* or student* or juvenile* or underage or young people or young person or young adult or girl or boy).mp
18	exp adolescence/
19	17 or 18
20	11 and 16 and 19

For peer review only

Cochrane Library

	Searches
1.	South Asia or Afghanistan or Bangladesh or Bhutan or India or Maldives or Nepal or Pakistan or Sri Lanka
2.	malnutrition or undernutrition or under nutrition or under-nutrition or stunting or stunted or underweight or under-weight or under weight or thinness or micronutrient deficienc*
3.	adolescen* or teen* or youth* or student* or juvenile* or underage or young people or young person or young adult or girl or boy
4	#1 AND #2 AND #3

Web of Science

	Searches
1.	TS=(South Asia or Afghanistan or Bangladesh or Bhutan or India or Maldives or Nepal or Pakistan or Sri Lanka)
2.	TS=(malnutrition or undernutrition or under nutrition or under-nutrition or stunting or stunted or underweight or under-weight or under weight or thinness or micronutrient deficienc*)
3.	TS=(adolescen* or teen* or youth* or student* or juvenile* or underage or young people or young person or young adult or girl or boy)
4.	#3 AND #2 AND #1

CINAHL

	Searches
1	"South Asia"
2	(MH "Asia, Western+")
3	"Afghanistan"
4	"Bangladesh"
5	"Bhutan"
6	"India"
7	"Maldives"
8	"Nepal"
9	"Pakistan"
10	"Sri Lanka"
11	S1 OR S2 OR S3 OR S4 OR S5 OR S6 OR S7 OR S8 OR S9 OR S10
12	(MH "Malnutrition") OR "malnutrition"
13	(MH "Thinness") OR "thinness"
14	"micronutrient deficiency" OR (MH "Vitamin B6 Deficiency") OR (MH "Vitamin B12 Deficiency") OR (MH "Folic Acid Deficiency") OR (MH "Ascorbic Acid Deficiency") OR (MH "Vitamin B Deficiency") OR (MH "Riboflavin Deficiency") OR (MH "Vitamin E Deficiency") OR (MH "Thiamine Deficiency") OR (MH "Vitamin D Deficiency") OR (MH "Vitamin A Deficiency")
15	"undernutrition"
16	"stunting"
17	S11 OR S12 OR S13
18	"adolescents or teenagers or young adults" OR (MH "Young Adult") OR (MH "Adolescence")
	S10 AND S14 AND S15

For peer review only

Psychinfo

	Searches
1	South Asia*.mp.
2	Afghanistan.mp.
3	Bangladesh.mp.
4	Bhutan.mp.
5	India.mp.
6	Maldives.mp.
7	Nepal.mp.
8	Pakistan.mp.
9	Sri Lanka.mp.
10	1 or 2 or 3 or 4 or 5 or 6 or 7 or 8 or 9
11	exp Nutritional Deficiencies/
12	(malnutrition or undernutrition or under nutrition or under-nutrition or stunting or stunted or underweight or under-weight or under weight or thinness or micronutrient deficienc*).mp. [mp=title, abstract, heading word, table of contents, key concepts, original title, tests & measures]
13	11 or 12
14	(adolescen* or teen* or youth* or student* or juvenile* or underage or young people or young person or young adult or girl or boy).mp
15	10 and 13 and 14

Scopus

((TITLE-ABS-KEY (afghanistan) OR TITLE-ABS-KEY (bangladesh) OR TITLE-ABS-KEY (bhutan) OR TITLE-ABS-KEY (india) OR TITLE-ABS-KEY (maldives) OR TITLE-ABS-KEY (nepal) OR TITLE-ABS-KEY (pakistan) OR TITLE-ABS-KEY (sri AND lanka) OR TITLE-ABS-KEY (south AND asia*))) AND ((TITLE-ABS-KEY (youth*) OR TITLE-ABS-KEY (adolescen*) OR TITLE-ABS-KEY (student*) OR TITLE-ABS-KEY (juvenile*) OR TITLE-ABS-KEY (underage) OR TITLE-ABS-KEY (young AND people) OR TITLE-ABS-KEY (young AND person) OR TITLE-ABS-KEY (young AND adult) OR TITLE-ABS-KEY (girl) OR TITLE-ABS-KEY (boy) OR TITLE-ABS-KEY (teen*))) AND ((TITLE-ABS-KEY (malnutrition) OR TITLE-ABS-KEY (undernutrition) OR TITLE-ABS-KEY (under AND nutrition) OR TITLE-ABS-KEY (under-nutrition) OR TITLE-ABS-KEY (stunting) OR TITLE-ABS-KEY (stunted) OR TITLE-ABS-KEY (underweight) OR TITLE-ABS-KEY (under-weight) OR TITLE-ABS-KEY (under AND weight) OR TITLE-ABS-KEY (thinness) OR TITLE-ABS-KEY (micronutrient AND deficienc*))) AND (EXCLUDE (SUBJAREA , "BIOC") OR EXCLUDE (SUBJAREA , "ENVI") OR EXCLUDE (SUBJAREA , "IMMU") OR EXCLUDE (SUBJAREA , "PHAR") OR EXCLUDE (SUBJAREA , "ARTS") OR EXCLUDE (SUBJAREA , "ECON") OR EXCLUDE (SUBJAREA , "PSYC") OR EXCLUDE (SUBJAREA , "ENGI") OR EXCLUDE (SUBJAREA , "NEUR") OR EXCLUDE (SUBJAREA , "DENT") OR EXCLUDE (SUBJAREA , "EART") OR EXCLUDE (SUBJAREA , "CHEM") OR EXCLUDE (SUBJAREA , "ENER") OR EXCLUDE (SUBJAREA , "CENG") OR EXCLUDE (SUBJAREA , "MATH") OR EXCLUDE (SUBJAREA , "VETE") OR EXCLUDE (SUBJAREA , "BUSI") OR EXCLUDE (SUBJAREA , "DECI") OR EXCLUDE (SUBJAREA , "COMP") OR EXCLUDE (SUBJAREA , "MATE") OR EXCLUDE (SUBJAREA , "PHYS") OR EXCLUDE (SUBJAREA , "Undefined"))

For peer review only

Grey literature sources

WHOLIS – the WHO Library Information System	http://kohahq.searo.who.int/
eLENA e-Library of Evidence for Nutrition Actions	https://www.who.int/elena/en/
Opengrey	http://www.opengrey.eu/
WHO	https://www.who.int/en
UNICEF	https://www.unicef.org/
DHS program	https://dhsprogram.com/
Planning and Development Department AJ&K	https://pndajk.gov.pk/
GHDx	http://ghdx.healthdata.org/geography/pakistan
WFP	https://www1.wfp.org/
World Bank eLibrary	https://elibrary.worldbank.org/

For peer review only

BMJ Open

Adolescent undernutrition in South Asia: a scoping review protocol

Journal:	*BMJ Open*
Manuscript ID	bmjopen-2019-031955.R1
Article Type:	Protocol
Date Submitted by the Author:	06-Dec-2019
Complete List of Authors:	Estecha Querol, Sara; University of Warwick Warwick Medical School, Academic Unit of Primary Care; University of Warwick Warwick Medical School, NIHR Global Health Research Unit in Improving Health in Slums<br>Al-Khudairy, Lena; University of Warwick Warwick Medical School, Division of Health Sciences<br>Iqbal, Romaina; Aga Khan University, Department of Community Health Sciences and Medicine<br>Johnson, Samantha ; University of Warwick Warwick Medical School,<br>Gill, Paramjit ; University of Warwick Warwick Medical School, Academic Unit of Primary Care; University of Warwick Warwick Medical School, NIHR Global Health Research Unit in Improving Health in Slums
**Primary Subject Heading**:	Nutrition and metabolism
Secondary Subject Heading:	Global health
Keywords:	South Asia, Adolescents, undernutrition, Scoping review

SCHOLARONE™
Manuscripts

I, the Submitting Author has the right to grant and does grant on behalf of all authors of the Work (as defined in the below author licence), an exclusive licence and/or a non-exclusive licence for contributions from authors who are: i) UK Crown employees; ii) where BMJ has agreed a CC-BY licence shall apply, and/or iii) in accordance with the terms applicable for US Federal Government officers or employees acting as part of their official duties; on a worldwide, perpetual, irrevocable, royalty-free basis to BMJ Publishing Group Ltd ("BMJ") its licensees and where the relevant Journal is co-owned by BMJ to the co-owners of the Journal, to publish the Work in this journal and any other BMJ products and to exploit all rights, as set out in our licence.

The Submitting Author accepts and understands that any supply made under these terms is made by BMJ to the Submitting Author unless you are acting as an employee on behalf of your employer or a postgraduate student of an affiliated institution which is paying any applicable article publishing charge ("APC") for Open Access articles. Where the Submitting Author wishes to make the Work available on an Open Access basis (and intends to pay the relevant APC), the terms of reuse of such Open Access shall be governed by a Creative Commons licence – details of these licences and which Creative Commons licence will apply to this Work are set out in our licence referred to above.

Other than as permitted in any relevant BMJ Author's Self Archiving Policies, I confirm this Work has not been accepted for publication elsewhere, is not being considered for publication elsewhere and does not duplicate material already published. I confirm all authors consent to publication of this Work and authorise the granting of this licence.

For peer review only

TITLE PAGE

Title of the article:

Adolescent undernutrition in South Asia: a scoping review protocol

Full name, postal address, e-mail and telephone number of the corresponding author:

Sara Estecha Querol

Warwick Medical School, University of Warwick

Gibbet Hill Road, Coventry (UK) CV4 7AL

Sara.Estecha-Querol@warwick.ac.uk

+44 2476575343

Full name, department, institution, city and country of all co-authors:

Sara Estecha Querol[1,2], Lena Al-Khudairy[3], Romaina Iqbal[4], Samantha Johnson[5], Paramjit Gill[1,2].

[1] Academic Unit of Primary Care, University of Warwick, Coventry, UK

[2] NIHR Global Health Research Unit in Improving Health in Slums, University of Warwick, Coventry, UK

[3] Division of Health Sciences, Warwick Medical School, University of Warwick, Coventry, UK

[4] Department of Community Health Sciences, Aga Khan University, Karachi, Pakistan.

[5] The Library, University of Warwick, Coventry, UK

Word count (excluding title page, abstract, references, figures and tables):

2,189 words

For peer review only - http://bmjopen.bmj.com/site/about/guidelines.xhtml

TITLE

Adolescent undernutrition in South Asia: a scoping review protocol

ABSTRACT

Introduction: The aim of the protocol is to present the methodology of a scoping review that aims to synthesise evidence up to date on adolescent undernutrition in South Asia.

Methods and analysis: The proposed scoping review will be guided by Arksey and O'Malley's framework and The Joanna Briggs Institute Reviewers' Manual. The scoping review question, eligibility criteria and search strategy will be based on the Population, Concept and Context (PCC) strategy. We will conduct the search in electronic bibliographic databases (Medline (OVID), Embase, Cochrane Library, Web of Science, CINAHL, PsycInfo, Scopus) as well as various grey literature sources in order to synthesise and present the findings with descriptive statistics and a narrative description of both quantitative and qualitative evidence.

Dissemination and ethics: This protocol will accurately describe the proposed scoping review that will map the evidence on adolescent malnutrition in South Asia. The proposed review aims to gather published and unpublished literature to inform policy and healthcare organizations as well as identify future research priorities in South Asia. This study protocol does not require ethical approval.

KEYWORDS: South Asia, adolescents, undernutrition, scoping review.

ARTICLE SUMMARY

Strengths and limitations of this study

- This protocol will clearly describe the proposed methods to collect and synthesise evidence on adolescent malnutrition in South Asia.
- The proposed search strategy will be conducted in seven electronic databases.
- Grey literature sources will be also included in the proposed scoping review, such as government reports and organisation websites.
- This proposed scoping review describes the mapping process of all types of literature available, however quality assessment is not required in scoping reviews.

For peer review only - http://bmjopen.bmj.com/site/about/guidelines.xhtml

For peer review only

- This protocol will ensure transparency in methodology and reduce the likelihood of reviewing bias.

For peer review only

For peer review only - http://bmjopen.bmj.com/site/about/guidelines.xhtml

INTRODUCTION

The most recent Global Nutrition Report highlights the global burden of malnutrition and that it remains unacceptably high affecting every country in the world.(1) The World Health Assembly formulated six global nutrition targets aiming to tackle the burden of malnutrition worldwide by 2025.(2) These targets plan to decrease stunting and wasting in children, reduce anaemia in women, decrease the rates of low birth weight, increase the rates of exclusive breastfeeding in the first 6 months, and ensure that there is no increase in childhood overweight. Malnutrition refers to deficiencies, excesses or imbalances in a person's intake of energy and/or nutrients.(3) Focusing on early stages in life, the term malnutrition covers several conditions affecting child growth patterns such as stunting, wasting, underweight, micronutrient deficiencies and overweight/obesity. Stunting, or low height for age, is caused among other factors, by long-term insufficient nutrient intake, frequent infections and diseases. Wasting, or low weight for height, also indicates malnutrition prevalence among children under 5 years of age. While wasting is the result of acute significant food shortage and/or disease, stunting represents chronic malnutrition and the effects are largely irreversible. Underweight, or low weight for age, includes children under 5 with low weight for height (wasting) and low height for age (stunting) and considered a proxy indicator for undernutrition if data on wasting are not available. Micronutrient deficiencies or insufficiencies, also called "hidden hunger", indicate a lack of important vitamins and minerals. Stunting, wasting, underweight and micronutrient deficiencies constitute the term "undernutrition".(3) Overweight/obesity is another form of malnutrition that generally involves the development of non-communicable diseases. Overweight/obesity are attributed to the term "overnutrition".(3) However, grown reference data and indicators are different between children and adolescents. The World Health Organization (WHO) recommends the use of BMI for age (thinness and overweight) and height for age (stunting) to explore adolescent growth patterns.(4, 5) Stunting is defined as height-for-age <2 SDs below the WHO Child Growth Reference median. Overweight is defined as BMI-for-age >1 SD above the WHO Growth Reference median, and obesity is defined as >2 SDs above the WHO Growth Reference median. Thinness is defined as BMI-for-age <2 SDs below the WHO Growth Reference median.(5) The term thinness and underweight have been used interchangeably when making reference to adolescents' low BMI-for-age.(6, 7)

The *State of food security and nutrition in the world* reviewed the global prevalence of thinness among children aged 5–9 years and adolescents aged 10–19 years.(8) Thinness (BMI for age below 2 SD) in these age groups was associated with a higher risk of infectious diseases, delayed maturation, reduced muscular strength, work capacity and bone density later in life. In addition, thinness in adolescent girls

For peer review only - http://bmjopen.bmj.com/site/about/guidelines.xhtml

was associated with adverse pregnancy outcomes and intrauterine growth retardation. Globally, 10% of children aged 5–19 have a BMI-for-age below 2 SD. There are significant differences in the prevalence of thinness among children aged 5–19 years by region of the world. For instance, the prevalence is high at >15% in South Asian countries such as India, Afghanistan, Bangladesh, Bhutan, Nepal, Pakistan and Sri Lanka in comparison to <2% in Latin America and the Caribbean, Northern America, Europe and Oceania. The global prevalence of thinness remained fairly steady over the past decade.(8) The prevalence of stunting (height for age below 2 SD), thinness (BMI for age below 2 SD) and overweight (BMI for age above 1 SD) were globally estimated using data from the Global School-Based Student Health and Health Behavior in School-Aged Children surveys conducted in 57 low and middle income countries between 2003 and 2013, involving 129,276 adolescents aged 12 to 15 years.(9) Globally, the prevalence of stunting was 10.2%, thinness was 5.5% and overweight 21.4%. Particularly in Pakistan, the prevalence of stunting was 7.1%, thinness was 11.2% and overweight 6.4% in 2009. In India in 2007, the prevalence of stunting was 14.6%, thinness was 15.9% and overweight 11.1%. In Sri Lanka in 2008, the prevalence of stunting was 25.6%, thinness was 31.5% and overweight 4.8%.

There are around of 1.8 billion adolescents worldwide and the majority are clustered in low and middle income countries.(6) However, there is a gap in adolescent health data.(10) Whilst the large global health and nutrition surveys mainly collected household data from adolescent girls aged ≥ 15 years; the 10 -14 year old age group, unmarried adolescents, unschooled adolescents, and male adolescents have not been included in global health surveys.(10) Christian and Smith(6) highlighted the public health need to investigate adolescent nutrition deficiencies, growth and development.

Child and adolescent malnutrition in South Asia was previously reviewed.(11-14) (see a summary of reviews in Table 1). Some literature reviews(11, 13, 14) were focused on children, while Mak's systematic review(12) provided evidence on both children and adolescents. However, Mak's systematic review exclusively focused on studies examining the prevalence of underweight status in the last two decades. This scoping review protocol will include a larger pool of evidence by proposing a broader search strategy including micronutrient deficiencies as well as including grey literature without restrictions on date of publication.

	Akhtar 2016(11)	Mak 2012(12)	Pasricha 2010(13)	Khan 2007(14)	The proposed scoping review
Type of study	Literature review	Systematic review	Literature review	Literature review	Scoping review

For peer review only - http://bmjopen.bmj.com/site/about/guidelines.xhtml

Date of publication the studies included	Restrictions on date of publication not specified	1990 - 2010	Restrictions on date of publication not specified	Restrictions on date of publication not specified	Up to 2019
Context of the population	South Asia (India, Pakistan, Bangladesh, Sri Lanka and Nepal)	East Asia (China, Hong Kong, Japan, South Korea, and Taiwan), South Asia (Bangladesh, India, Indonesia, Malaysia, Nepal, Pakistan, Singapore, Thailand, and Vietnam), and West Asia (Bahrain and Iran).	South and South-East Asia (no countries are listed)	South Asia (no countries are listed)	South Asia (Afghanistan, Bangladesh, Bhutan, India, Maldives, Nepal, Pakistan, and Sri Lanka)
Age of population	Children under 5 years and school children (up to 12 years)	Children and adolescents	Children under 5 years	Children under 5 years	Adolescents 10 – 19 years
Concept of malnutrition	Undernutrition	Underweight	Undernutrition	Undernutrition	Undernutrition

Table 1: Reviews on child and adolescent malnutrition in South Asia.

REVIEW AIMS

The aim of this protocol is to describe the methodology of an up to date scoping review of the literature on adolescent undernutrition in South Asia and identifying gaps in knowledge. The proposed scoping review work could potentially be valuable to inform policy makers and healthcare organizations delivering adolescent health and nutrition actions in South Asia. It will constitute the first step in a larger research project aimed at improving adolescent health in Pakistan; these findings will inform the next quantitative and qualitative phases.

For peer review only - http://bmjopen.bmj.com/site/about/guidelines.xhtml

METHODS AND ANALYSIS

All study types will be included to ensure the inclusion of the majority of relevant literature.(15) The design of the proposed scoping review methodology was informed by Arksey and O'Malley's framework(15) and The Joanna Briggs Institute Reviewers' Manual.(16) Arksey and O'Malley's methodological framework includes five stages to conduct a scoping review.

Stage 1: identifying the research question

We will use the Population, Concept and Context (PCC) strategy to define the title, scoping review objective, scoping review question, and inclusion criteria.(16) Therefore, our main research question will address: adolescent undernutrition in South Asia, what do we know from the existing literature?

Stage 2: identifying relevant studies

At this stage, the reviewing team (SEQ, SJ, RI, LAK, PG) discussed the key words constituting the search strategy and the criteria for inclusion and exclusion of the studies in accordance with the PCC strategy.

Databases and search strategy

An experienced research librarian (SJ) and one reviewer (SEQ) developed the search strategy by testing the keywords, MESH terms and databases to search. Finally, the reviewing team discussed and determined the final search strategy. The search will be conducted using the following electronic bibliographic databases and grey literature sources: Medline (OVID), Embase, Cochrane, Web of Science, CINAHL, PsycInfo, Scopus, the WHO Library Information System (WHOLIS), eLENA e-Library of Evidence for Nutrition Actions, and Opengrey. The websites of agencies, academic institutions and technical bodies will be also searched: World Health Organisation (WHO), United Nations International Children's Emergency Fund (UNICEF), Demographic and Health Surveys (DHS), Program, Planning and Development Department AJ&K, Global Health Data Exchange (GHDx), World Food Program (WFP), and World Bank eLibrary. In addition, the topic field expert (RI) will be consulted to identify additional relevant grey literature sources. One reviewer (SEQ) will search additional resources by hand searching the references of included studies to identify further relevant evidence. This search strategy includes South Asia AND adolescents AND undernutrition. Related terms to undernutrition were also considered in the search strategy such as stunting, thinness, underweight, and micronutrient deficiency. The search strategy was tailored to the specific requirements of each database. No restrictions on language or date of publication will be made. See full search strategy in appendix 1.

For peer review only - http://bmjopen.bmj.com/site/about/guidelines.xhtml

Eligibility criteria

Despite the broadness of the proposed scoping review research question, the eligibly criteria should be clearly defined in order to thoroughly guide the reviewers' decisions. The inclusion criteria listed below are based upon the PCC strategy:

- Population: adolescents mean age 10 – 19 years old.(17)
- Context: South Asia. The World Bank limits the South Asia region to Afghanistan, Bangladesh, Bhutan, India, Maldives, Nepal, Pakistan, and Sri Lanka.(18)
- Concept: the term malnutrition includes both under and over nutrition. However, in this proposed scoping review, malnutrition will refer only to undernutrition indicators used in adolescents (stunting, thinness or underweight, and micronutrient deficiencies(3)). Adolescent malnutrition will be assessed quantitatively using the following WHO indicators: 1) thinness or underweight (low BMI-for-age); 2) stunting (low height-for-age); and 3) micronutrient deficiencies. All studies will be included regardless of the growth references or cut-off values for micronutrients deficiency followed.
- Adolescent malnutrition in South Asia will be assessed also qualitatively, therefore qualitative studies exploring perspectives, experiences or opinions around adolescent undernutrition will be included.
- Type of studies: quantitative and qualitative studies as well as grey literature, e.g. primary research studies, reviews, government reports and guidelines.

Studies will be excluded if they include:

- Only overnutrition indicators (obesity and overweight).
- Pregnant or breastfeeding adolescents.
- Adolescent athletes.
- Adolescents with long term conditions such as diabetes, tuberculosis or HIV.
- Hospitalised adolescents.
- Intervention studies targeting treatment of a specific illness or condition such as diarrhoea.

Stage 3: study selection

The screening process of this proposed scoping review will comprise two phases. First, titles and abstract will be reviewed by two independent reviewers following a broad inclusion criteria, that is, studies looking at adolescent undernutrition in South Asia. Papers identified by either or both reviewers will be included in the next phase, which is the full-text screening. The same reviewers will

For peer review only - http://bmjopen.bmj.com/site/about/guidelines.xhtml

screen full-text studies using the eligibility criteria mentioned above. Disagreements will be resolved by either discussion or referral to a third reviewer. PRISMA flow diagram (see figure 1) will be presented to reflect the search process.

Figure 1: PRISMA Flow Diagram for scoping review process. Retrieved from Peters M, Godfrey C, McInerney P, Soares C, Khalil H, Parker D (2015) The Joanna Briggs Institute reviewers' manual 2015: methodology for JBI scoping reviews.(16)

Stage 4: charting the data

Key characteristics to be extracted from the included studies will be extracted using a pre-defined data extraction sheet. At this stage, one reviewer (SEQ) will conduct data extraction consisting of a summary table recording the key information of the selected studies (see Table 2).

Study details
• Author(s) / Organization<br>• Year of publication<br>• Country<br>• Province and city (where the study was published or conducted)<br>• Study design<br>• Aims<br>• Sample size
Data relevant to the PCC term
• Population: target population (age, sex)<br>• Context: country<br>• Concept: outcome quantitative measures (stunting, thinness or underweight, and micronutrient deficiencies). Also, note the growth references or the cut-off values for micronutrients deficiency.<br>• Concept: qualitative outcomes (experiences, opinions and perspectives).<br>• Summary of findings.<br>• Recommendations of the author(s)

Table 2: Key information to be extracted.

Stage 5: collating, summarising and reporting the results

The results of the review will be reported as a map of the data extracted in a tabular form showing descriptive statistics. The tables may display the results as distribution of studies per year,

For peer review only - http://bmjopen.bmj.com/site/about/guidelines.xhtml

geographical area where the study was conducted, location (community, hospital or school based), target population, outcomes, and methods. In addition, a narrative description of both quantitative and qualitative results will accompany the descriptive presentation of the results by grouping the data into meaningful summaries for better exposition of the findings. For the qualitative studies, thematic analysis will be used to analyse the findings if data permits. Data will be exported into Nvivo and an inductive coding approach will be followed.

Patient and Public Involvement

No patient involved.

DISSEMINATION AND ETHICS

This protocol will provides a high quality overview of the broad literature available on the adolescent malnutrition in South Asia. Thus, the proposed scoping review will methodologically map the evidence on this topic to identify research priorities in South Asia. The proposed review will also facilitate the identification of uncovered disparities among adolescents living in South Asia. The potential gaps could help policy makers and healthcare organizations to design new research questions as well as interventions to improve adolescent malnutrition in South Asia.

Since a scoping review involves a methodically integration and presentation of available resources, this study does not require ethics approval.

AUTHOR CONTRIBUTIONS

SEQ conceived the idea, developed the methods and wrote the first draft of the manuscript. PG, LAK and RI supervised the protocol, contributed to methods, and supported the drafting and editing the manuscript. SJ contributed meaningfully to design the search strategy (appendix 1). All authors revised and approved the final manuscript.

FUNDING STATEMENT

This work was supported by NIHR grant number 16/136/87. The research was commissioned by the National Institute of Health Research using Official Development Assistance (ODA) funding. The views expressed are those of the author(s) and not necessarily those of the NHS, the NIHR or the Department of Health and Social Care.

For peer review only - http://bmjopen.bmj.com/site/about/guidelines.xhtml

For peer review only

COMPLETING INTERESTS

None declared.

REFERENCES

1. Independent Expert Group of the Global Nutrition Report. 2018 Global Nutrition Report. 2018.
2. WHO. Global nutrition targets 2025: policy brief series (WHO/NMH/NHD/14.2). Geneva; 2014.
3. WHO. What is malnutrition? 2016 [Available from: http://www.who.int/features/qa/malnutrition/en/.
4. WHO. Growth reference data for 5-19 years 2007 [Available from: https://www.who.int/growthref/en/.
5. de Onis M, Onyango AW, Borghi E, Siyam A, Nishida C, Siekmann J. Development of a WHO growth reference for school-aged children and adolescents. Bulletin of the World Health Organization. 2007;85(9):660-7.
6. Christian P, Smith ER. Adolescent Undernutrition: Global Burden, Physiology, and Nutritional Risks. Annals of nutrition & metabolism. 2018;72(4):316-28.
7. NCD Risk Factor Collaboration. Worldwide trends in body-mass index, underweight, overweight, and obesity from 1975 to 2016: a pooled analysis of 2416 population-based measurement studies in 128.9 million children, adolescents, and adults. Lancet (London, England). 2017;390(10113):2627-42.
8. FAO, IFAD, UNICEF, WFP, WHO. The State of Food Security and Nutrition in the World 2018. Building climate resilience for food security and nutrition. FAO; 2018.
9. Caleyachetty R, Thomas GN, Kengne AP, Echouffo-Tcheugui JB, Schilsky S, Khodabocus J, et al. The double burden of malnutrition among adolescents: analysis of data from the Global School-Based Student Health and Health Behavior in School-Aged Children surveys in 57 low- and middle-income countries. American Journal of Clinical Nutrition. 2018;108(2):414-24.
10. Mokdad AH, Forouzanfar MH, Daoud F, Mokdad AA, El Bcheraoui C, Moradi-Lakeh M, et al. Global burden of diseases, injuries, and risk factors for young people's health during 1990-2013: a systematic analysis for the Global Burden of Disease Study 2013. Lancet (London, England). 2016;387(10036):2383-401.
11. Akhtar S. Malnutrition in South Asia-A Critical Reappraisal. Critical reviews in food science and nutrition. 2016;56(14):2320-30.
12. Mak KK, Tan SH. Underweight problems in Asian children and adolescents. European journal of pediatrics. 2012;171(5):779-85.
13. Pasricha S-R, Biggs B-A. Undernutrition among children in South and South-East Asia. J Paediatr Child Health. 2010;46(9):497-503.
14. Khan AA, Bano N, Salam A. Child Malnutrition in South Asia: A Comparative Perspective. South Asian Survey. 2007;14(1):129-45.
15. Arksey H, O'Malley L. Scoping studies: towards a methodological framework. International Journal of Social Research Methodology. 2005;8(1):19-32.
16. Peters M, Godfrey C, McInerney P, Soares C, Khalil H, Parker D. The Joanna Briggs Institute reviewers' manual 2015: methodology for JBI scoping reviews. Adelaide, SA Australia: The Joanna Briggs Institute; 2015. 24 p.
17. WHO. Adolescent health and development 2019 [Available from: http://www.searo.who.int/entity/child_adolescent/topics/adolescent_health/en/.

For peer review only - http://bmjopen.bmj.com/site/about/guidelines.xhtml

18. The World Bank. The World Bank in South Asia 2019 [Available from: http://www.worldbank.org/en/region/sar/overview.

For peer review only

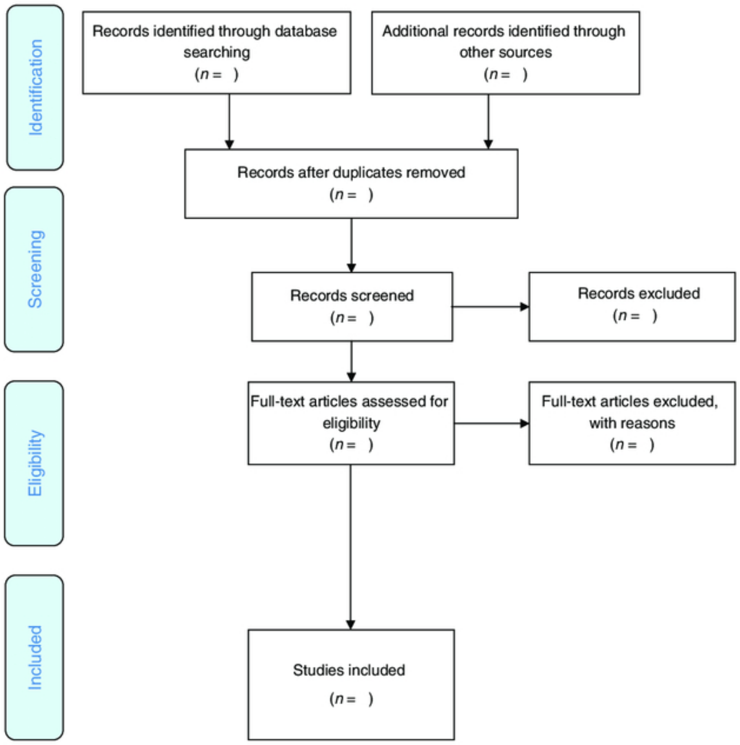

Figure 1: PRISMA Flow Diagram for scoping review process. Retrieved from Peters M, Godfrey C, McInerney P, Soares C, Khalil H, Parker D (2015) The Joanna Briggs Institute reviewers' manual 2015: methodology for JBI scoping reviews.(16)

299x251mm (72 x 72 DPI)

Appendix 1: search strategy

Medline: ovid

	Searches
1	South Asia*.mp.
2	South East Asia*.mp.
3	exp Afghanistan/
4	exp Bangladesh/
5	exp Bhutan/
6	exp India/
7	Maldives.mp.
8	exp Nepal/
9	exp Pakistan/
10	exp Sri Lanka/
11	1 or 2 or 3 or 4 or 5 or 6 or 7 or 8 or 9 or 10
12	exp Thinness/
13	exp malnutrition/ or exp deficiency diseases/
14	(undernutrition or under nutrition or under-nutrition or stunting or stunted or underweight or under-weight or under weight or thinness or micronutrient deficienc*).mp. [mp=title, abstract, original title, name of substance word, subject heading word, floating sub-heading word, keyword heading word, organism supplementary concept word, protocol supplementary concept word, rare disease supplementary concept word, unique identifier, synonyms]
15	12 or 13 or 14
16	(adolescen* or teen* or youth* or student* or juvenile* or underage or young people or young person or young adult or girl or boy).mp
17	exp Adolescent/
18	16 or 17
19	11 and 15 and 18

Embase: ovid

	Searches
1	exp South Asia/
2	exp Southeastern Asia/
3	exp Afghanistan/
4	exp Bangladesh/
5	exp Bhutan/
6	exp India/
7	exp Maldives/
8	exp Nepal/
9	exp Pakistan/
10	exp Sri Lanka/
11	1 or 2 or 3 or 4 or 5 or 6 or 7 or 8 or 9 or 10
12	exp malnutrition/
13	exp underweight/
14	exp nutritional deficiency/
15	(undernutrition or under nutrition or under-nutrition or stunting or stunted or underweight or under-weight or under weight or thinness or micronutrient deficienc*).mp. [mp=title, abstract, heading word, drug trade name, original title, device manufacturer, drug manufacturer, device trade name, keyword, floating subheading word, candidate term word]
16	12 or 13 or 14 or 15
17	(adolescen* or teen* or youth* or student* or juvenile* or underage or young people or young person or young adult or girl or boy).mp
18	exp adolescence/
19	17 or 18
20	11 and 16 and 19

For peer review only

Cochrane Library

	Searches
1.	South Asia or Afghanistan or Bangladesh or Bhutan or India or Maldives or Nepal or Pakistan or Sri Lanka
2.	malnutrition or undernutrition or under nutrition or under-nutrition or stunting or stunted or underweight or under-weight or under weight or thinness or micronutrient deficienc*
3.	adolescen* or teen* or youth* or student* or juvenile* or underage or young people or young person or young adult or girl or boy
4	#1 AND #2 AND #3

Web of Science

	Searches
1.	TS=(South Asia or Afghanistan or Bangladesh or Bhutan or India or Maldives or Nepal or Pakistan or Sri Lanka)
2.	TS=(malnutrition or undernutrition or under nutrition or under-nutrition or stunting or stunted or underweight or under-weight or under weight or thinness or micronutrient deficienc*)
3.	TS=(adolescen* or teen* or youth* or student* or juvenile* or underage or young people or young person or young adult or girl or boy)
4.	#3 AND #2 AND #1

CINAHL

	Searches
1	“South Asia”
2	(MH “Asia, Western+”)
3	“Afghanistan”
4	“Bangladesh”
5	“Bhutan”
6	“India”
7	“Maldives”
8	“Nepal”
9	“Pakistan”
10	“Sri Lanka”
11	S1 OR S2 OR S3 OR S4 OR S5 OR S6 OR S7 OR S8 OR S9 OR S10
12	(MH "Malnutrition") OR "malnutrition"
13	(MH "Thinness") OR "thinness"
14	"micronutrient deficiency" OR (MH "Vitamin B6 Deficiency") OR (MH "Vitamin B12 Deficiency") OR (MH "Folic Acid Deficiency") OR (MH "Ascorbic Acid Deficiency") OR (MH "Vitamin B Deficiency") OR (MH "Riboflavin Deficiency") OR (MH "Vitamin E Deficiency") OR (MH "Thiamine Deficiency") OR (MH "Vitamin D Deficiency") OR (MH "Vitamin A Deficiency")
15	"undernutrition"
16	"stunting"
17	S11 OR S12 OR S13
18	"adolescents or teenagers or young adults" OR (MH "Young Adult") OR (MH "Adolescence")
	S10 AND S14 AND S15

For peer review only

Psychinfo

	Searches
1	South Asia*.mp.
2	Afghanistan.mp.
3	Bangladesh.mp.
4	Bhutan.mp.
5	India.mp.
6	Maldives.mp.
7	Nepal.mp.
8	Pakistan.mp.
9	Sri Lanka.mp.
10	1 or 2 or 3 or 4 or 5 or 6 or 7 or 8 or 9
11	exp Nutritional Deficiencies/
12	(malnutrition or undernutrition or under nutrition or under-nutrition or stunting or stunted or underweight or under-weight or under weight or thinness or micronutrient deficienc*).mp. [mp=title, abstract, heading word, table of contents, key concepts, original title, tests & measures]
13	11 or 12
14	(adolescen* or teen* or youth* or student* or juvenile* or underage or young people or young person or young adult or girl or boy).mp
15	10 and 13 and 14

Scopus

((TITLE-ABS-KEY (afghanistan) OR TITLE-ABS-KEY (bangladesh) OR TITLE-ABS-KEY (bhutan) OR TITLE-ABS-KEY (india) OR TITLE-ABS-KEY (maldives) OR TITLE-ABS-KEY (nepal) OR TITLE-ABS-KEY (pakistan) OR TITLE-ABS-KEY (sri AND lanka) OR TITLE-ABS-KEY (south AND asia*))) AND ((TITLE-ABS-KEY (youth*) OR TITLE-ABS-KEY (adolescen*) OR TITLE-ABS-KEY (student*) OR TITLE-ABS-KEY (juvenile*) OR TITLE-ABS-KEY (underage) OR TITLE-ABS-KEY (young AND people) OR TITLE-ABS-KEY (young AND person) OR TITLE-ABS-KEY (young AND adult) OR TITLE-ABS-KEY (girl) OR TITLE-ABS-KEY (boy) OR TITLE-ABS-KEY (teen*))) AND ((TITLE-ABS-KEY (malnutrition) OR TITLE-ABS-KEY (undernutrition) OR TITLE-ABS-KEY (under AND nutrition) OR TITLE-ABS-KEY (under-nutrition) OR TITLE-ABS-KEY (stunting) OR TITLE-ABS-KEY (stunted) OR TITLE-ABS-KEY (underweight) OR TITLE-ABS-KEY (under-weight) OR TITLE-ABS-KEY (under AND weight) OR TITLE-ABS-KEY (thinness) OR TITLE-ABS-KEY (micronutrient AND deficienc*))) AND (EXCLUDE (SUBJAREA , "BIOC") OR EXCLUDE (SUBJAREA , "ENVI") OR EXCLUDE (SUBJAREA , "IMMU") OR EXCLUDE (SUBJAREA , "PHAR") OR EXCLUDE (SUBJAREA , "ARTS") OR EXCLUDE (SUBJAREA , "ECON") OR EXCLUDE (SUBJAREA , "PSYC") OR EXCLUDE (SUBJAREA , "ENGI") OR EXCLUDE (SUBJAREA , "NEUR") OR EXCLUDE (SUBJAREA , "DENT") OR EXCLUDE (SUBJAREA , "EART") OR EXCLUDE (SUBJAREA , "CHEM") OR EXCLUDE (SUBJAREA , "ENER") OR EXCLUDE (SUBJAREA , "CENG") OR EXCLUDE (SUBJAREA , "MATH") OR EXCLUDE (SUBJAREA , "VETE") OR EXCLUDE (SUBJAREA , "BUSI") OR EXCLUDE (SUBJAREA , "DECI") OR EXCLUDE (SUBJAREA , "COMP") OR EXCLUDE (SUBJAREA , "MATE") OR EXCLUDE (SUBJAREA , "PHYS") OR EXCLUDE (SUBJAREA , "Undefined"))

For peer review only

Grey literature sources

WHOLIS – the WHO Library Information System	http://kohahq.searo.who.int/
eLENA e-Library of Evidence for Nutrition Actions	https://www.who.int/elena/en/
Opengrey	http://www.opengrey.eu/
WHO	https://www.who.int/en
UNICEF	https://www.unicef.org/
DHS program	https://dhsprogram.com/
Planning and Development Department AJ&K	https://pndajk.gov.pk/
GHDx	http://ghdx.healthdata.org/geography/pakistan
WFP	https://www1.wfp.org/
World Bank eLibrary	https://elibrary.worldbank.org/

For peer review only

BMJ Open

Adolescent undernutrition in South Asia: a scoping review protocol

Journal:	*BMJ Open*
Manuscript ID	bmjopen-2019-031955.R2
Article Type:	Protocol
Date Submitted by the Author:	25-Dec-2019

Complete List of Authors:	Estecha Querol, Sara; University of Warwick Warwick Medical School, Academic Unit of Primary Care; University of Warwick Warwick Medical School, NIHR Global Health Research Unit in Improving Health in Slums<br>Al-Khudairy, Lena; University of Warwick Warwick Medical School, Division of Health Sciences<br>Iqbal, Romaina; Aga Khan University, Department of Community Health Sciences and Medicine<br>Johnson, Samantha ; University of Warwick Warwick Medical School,<br>Gill, Paramjit ; University of Warwick Warwick Medical School, Academic Unit of Primary Care; University of Warwick Warwick Medical School, NIHR Global Health Research Unit in Improving Health in Slums

**Primary Subject Heading**:	Nutrition and metabolism
Secondary Subject Heading:	Global health
Keywords:	South Asia, Adolescents, undernutrition, Scoping review

SCHOLARONE™
Manuscripts

I, the Submitting Author has the right to grant and does grant on behalf of all authors of the Work (as defined in the below author licence), an exclusive licence and/or a non-exclusive licence for contributions from authors who are: i) UK Crown employees; ii) where BMJ has agreed a CC-BY licence shall apply, and/or iii) in accordance with the terms applicable for US Federal Government officers or employees acting as part of their official duties; on a worldwide, perpetual, irrevocable, royalty-free basis to BMJ Publishing Group Ltd ("BMJ") its licensees and where the relevant Journal is co-owned by BMJ to the co-owners of the Journal, to publish the Work in this journal and any other BMJ products and to exploit all rights, as set out in our licence.

The Submitting Author accepts and understands that any supply made under these terms is made by BMJ to the Submitting Author unless you are acting as an employee on behalf of your employer or a postgraduate student of an affiliated institution which is paying any applicable article publishing charge ("APC") for Open Access articles. Where the Submitting Author wishes to make the Work available on an Open Access basis (and intends to pay the relevant APC), the terms of reuse of such Open Access shall be governed by a Creative Commons licence – details of these licences and which Creative Commons licence will apply to this Work are set out in our licence referred to above.

Other than as permitted in any relevant BMJ Author's Self Archiving Policies, I confirm this Work has not been accepted for publication elsewhere, is not being considered for publication elsewhere and does not duplicate material already published. I confirm all authors consent to publication of this Work and authorise the granting of this licence.

For peer review only

TITLE PAGE

Title of the article:

Adolescent undernutrition in South Asia: a scoping review protocol

Full name, postal address, e-mail and telephone number of the corresponding author:

Sara Estecha Querol

Warwick Medical School, University of Warwick

Gibbet Hill Road, Coventry (UK) CV4 7AL

Sara.Estecha-Querol@warwick.ac.uk

+44 2476575343

Full name, department, institution, city and country of all co-authors:

Sara Estecha Querol[1,2], Lena Al-Khudairy[3], Romaina Iqbal[4], Samantha Johnson[5], Paramjit Gill[1,2].

[1] Academic Unit of Primary Care, University of Warwick, Coventry, UK

[2] NIHR Global Health Research Unit in Improving Health in Slums, University of Warwick, Coventry, UK

[3] Division of Health Sciences, Warwick Medical School, University of Warwick, Coventry, UK

[4] Department of Community Health Sciences, Aga Khan University, Karachi, Pakistan.

[5] The Library, University of Warwick, Coventry, UK

Word count (excluding title page, abstract, references, figures and tables):

2,189 words

For peer review only - http://bmjopen.bmj.com/site/about/guidelines.xhtml

TITLE

Adolescent undernutrition in South Asia: a scoping review protocol

ABSTRACT

Introduction: The aim of the protocol is to present the methodology of a scoping review that aims to synthesise up-to-date evidence on adolescent undernutrition in South Asia.

Methods and analysis: The proposed scoping review will be guided by Arksey and O'Malley's framework and the Joanna Briggs Institute Reviewers' Manual. The scoping review question, eligibility criteria and search strategy will be based on the Population, Concept and Context (PCC) strategy. We will conduct the search in electronic bibliographic databases (Medline (OVID), Embase, Cochrane Library, Web of Science, CINAHL, PsycInfo, Scopus) as well as various grey literature sources in order to synthesise and present the findings with descriptive statistics and a narrative description of both quantitative and qualitative evidence.

Dissemination and ethics: This protocol will accurately describe the proposed scoping review that will map the evidence on adolescent undernutrition in South Asia. The proposed review aims to gather published and unpublished literature to inform policy and healthcare organizations as well as identify future research priorities in South Asia. This study protocol does not require ethical approval.

KEYWORDS: South Asia, adolescents, undernutrition, scoping review.

Strengths and limitations of this study

- This protocol will ensure transparency in methodology and reduce the likelihood of reviewing bias.
- This protocol will clearly describe the proposed methods to collect and synthesise evidence on adolescent undernutrition in South Asia.
- The proposed search strategy will be conducted in seven electronic databases.
- Grey literature sources will be also included in the proposed scoping review, such as government reports and organisation websites.
- There will be no quality assessment of the included studies.

For peer review only - http://bmjopen.bmj.com/site/about/guidelines.xhtml

For peer review only

For peer review only

For peer review only - http://bmjopen.bmj.com/site/about/guidelines.xhtml

INTRODUCTION

The most recent Global Nutrition Report highlights the global burden of malnutrition and that it remains unacceptably high affecting every country in the world.(1) The World Health Assembly formulated six global nutrition targets aiming to tackle the burden of malnutrition worldwide by 2025.(2) These targets plan to decrease stunting and wasting in children, reduce anaemia in women, decrease the rates of low birth weight, increase the rates of exclusive breastfeeding in the first 6 months, and ensure that there is no increase in childhood overweight. Malnutrition refers to deficiencies, excesses or imbalances in a person's intake of energy and/or nutrients.(3) Focusing on early stages in life, the term malnutrition covers several conditions affecting child growth patterns such as stunting, wasting, underweight, micronutrient deficiencies and overweight/obesity. Stunting, or low height for age, is caused among other factors, by long-term insufficient nutrient intake, frequent infections and diseases. Wasting, or low weight for height, also indicates malnutrition prevalence among children under 5 years of age. While wasting is the result of acute significant food shortage and/or disease, stunting represents chronic malnutrition and the effects are largely irreversible. Underweight, or low weight for age, includes children under 5 with low weight for height (wasting) and low height for age (stunting) and considered a proxy indicator for undernutrition if data on wasting is not available. Micronutrient deficiencies or insufficiencies, also called "hidden hunger", indicate a lack of important vitamins and minerals. Stunting, wasting, underweight and micronutrient deficiencies constitute the term "undernutrition".(3) Overweight/obesity is another form of malnutrition that generally involves the development of non-communicable diseases. Overweight/obesity are attributed to the term "overnutrition".(3) However, grown reference data and indicators are different between children and adolescents. The World Health Organization (WHO) recommends the use of BMI for age (thinness and overweight) and height for age (stunting) to explore adolescent growth patterns.(4, 5) Stunting is defined as height-for-age <2 SDs below the WHO Child Growth Reference median. Overweight is defined as BMI-for-age >1 SD above the WHO Growth Reference median, and obesity is defined as >2 SDs above the WHO Growth Reference median. Thinness is defined as BMI-for-age <2 SDs below the WHO Growth Reference median.(5) The term thinness and underweight have been used interchangeably when making reference to adolescents' low BMI-for-age.(6, 7)

The *State of food security and nutrition in the world* reviewed the global prevalence of thinness among children aged 5–9 years and adolescents aged 10–19 years.(8) Thinness (BMI for age below 2 SD) in these age groups was associated with a higher risk of infectious diseases, delayed maturation, reduced muscular strength, work capacity and bone density later in life. In addition, thinness in

For peer review only - http://bmjopen.bmj.com/site/about/guidelines.xhtml

adolescent girls was associated with adverse pregnancy outcomes and intrauterine growth retardation. Globally, 10% of children aged 5–19 have a BMI-for-age below 2 SD. There are significant differences in the prevalence of thinness among children aged 5–19 years by region of the world. For instance, the prevalence is high at >15% in South Asian countries such as India, Afghanistan, Bangladesh, Bhutan, Nepal, Pakistan and Sri Lanka in comparison to <2% in Latin America and the Caribbean, Northern America, Europe and Oceania. The global prevalence of thinness remained fairly steady over the past decade.(8) The prevalence of stunting (height for age below 2 SD), thinness (BMI for age below 2 SD) and overweight (BMI for age above 1 SD) were globally estimated using data from the Global School-Based Student Health and Health Behavior in School-Aged Children surveys conducted in 57 low and middle income countries between 2003 and 2013, involving 129,276 adolescents aged 12 to 15 years.(9) Globally, the prevalence of stunting was 10.2%, thinness was 5.5% and overweight 21.4%. Particularly in Pakistan, the prevalence of stunting was 7.1%, thinness was 11.2% and overweight 6.4% in 2009. In India in 2007, the prevalence of stunting was 14.6%, thinness was 15.9% and overweight 11.1%. In Sri Lanka in 2008, the prevalence of stunting was 25.6%, thinness was 31.5% and overweight 4.8%.

There are around of 1.8 billion adolescents worldwide and the majority are clustered in low and middle income countries.(6) However, there is a gap in adolescent health data.(10) Whilst the large global health and nutrition surveys mainly collected household data from adolescent girls aged ≥ 15 years; the 10 -14 year old age group, unmarried adolescents, unschooled adolescents, and male adolescents have not been included in global health surveys.(10) Christian and Smith(6) highlighted the public health need to investigate adolescent nutrition deficiencies, growth and development.

Child and adolescent undernutrition in South Asia was previously reviewed.(11-14) (see a summary of reviews in Table 1). Some literature reviews(11, 13, 14) were focused on children, while Mak's systematic review(12) provided evidence on both children and adolescents. However, Mak's systematic review exclusively focused on studies examining the prevalence of underweight status in the last two decades. This scoping review protocol will include a larger pool of evidence by proposing a broader search strategy including micronutrient deficiencies as well as including grey literature without restrictions on date of publication.

	Akhtar 2016(11)	Mak 2012(12)	Pasricha 2010(13)	Khan 2007(14)	The proposed scoping review
Type of study	Literature review	Systematic review	Literature review	Literature review	Scoping review
Date of	Restrictions on	1990 - 2010	Restrictions on	Restrictions on	Up to 2019

For peer review only - http://bmjopen.bmj.com/site/about/guidelines.xhtml

publication the studies included	date of publication not specified		date of publication not specified	date of publication not specified	
Context of the population	South Asia (India, Pakistan, Bangladesh, Sri Lanka and Nepal)	East Asia (China, Hong Kong, Japan, South Korea, and Taiwan), South Asia (Bangladesh, India, Indonesia, Malaysia, Nepal, Pakistan, Singapore, Thailand, and Vietnam), and West Asia (Bahrain and Iran).	South and South-East Asia (no countries are listed)	South Asia (no countries are listed)	South Asia (Afghanistan, Bangladesh, Bhutan, India, Maldives, Nepal, Pakistan, and Sri Lanka)
Age of population	Children under 5 years and school children (up to 12 years)	Children and adolescents	Children under 5 years	Children under 5 years	Adolescents 10 – 19 years
Concept of malnutrition	Undernutrition	Underweight	Undernutrition	Undernutrition	Undernutrition

Table 1: Reviews on child and adolescent undernutrition in South Asia.

REVIEW AIMS

The aim of this protocol is to describe the methodology of an up-to-date scoping review of the literature on adolescent undernutrition in South Asia and identifying gaps in knowledge. The proposed scoping review work could potentially be valuable to inform policy makers and healthcare organizations delivering adolescent health and nutrition actions in South Asia. It will constitute the first step in a larger research project aimed at improving adolescent health in Pakistan; these findings will inform the next quantitative and qualitative phases.

METHODS AND ANALYSIS

All study types will be included to ensure the inclusion of the majority of relevant literature.(15) The design of the proposed scoping review methodology was informed by Arksey and O'Malley's

For peer review only - http://bmjopen.bmj.com/site/about/guidelines.xhtml

framework(15) and The Joanna Briggs Institute Reviewers' Manual.(16) Arksey and O'Malley's methodological framework includes five stages to conduct a scoping review.

Stage 1: identifying the research question

We will use the Population, Concept and Context (PCC) strategy to define the title, scoping review objective, scoping review question, and inclusion criteria.(16) Therefore, our main research question will address: adolescent undernutrition in South Asia, what do we know from the existing literature?

Stage 2: identifying relevant studies

At this stage, the reviewing team (SEQ, SJ, RI, LAK, PG) discussed the key words constituting the search strategy and the criteria for inclusion and exclusion of the studies in accordance with the PCC strategy.

Databases and search strategy

An experienced research librarian (SJ) and one reviewer (SEQ) developed the search strategy by testing the keywords, MESH terms and databases to search. Finally, the reviewing team discussed and determined the final search strategy. The search will be conducted using the following electronic bibliographic databases and grey literature sources: Medline (OVID), Embase, Cochrane, Web of Science, CINAHL, PsycInfo, Scopus, the WHO Library Information System (WHOLIS), eLENA e-Library of Evidence for Nutrition Actions, and Opengrey. The websites of agencies, academic institutions and technical bodies will be also be searched: World Health Organisation (WHO), United Nations International Children's Emergency Fund (UNICEF), Demographic and Health Surveys (DHS), Program, Planning and Development Department AJ&K, Global Health Data Exchange (GHDx), World Food Program (WFP), and World Bank eLibrary. In addition, the topic field expert (RI) will be consulted to identify additional relevant grey literature sources. One reviewer (SEQ) will search additional resources by hand searching the references of included studies to identify further relevant evidence. This search strategy includes South Asia AND adolescents AND undernutrition. Related terms to undernutrition were also considered in the search strategy such as stunting, thinness, underweight, and micronutrient deficiency. The search strategy was tailored to the specific requirements of each database. No restrictions on language or date of publication will be made. See full search strategy in appendix 1.

For peer review only - http://bmjopen.bmj.com/site/about/guidelines.xhtml

Eligibility criteria

Despite the broadness of the proposed scoping review research question, the elegibility criteria should be clearly defined in order to thoroughly guide the reviewers' decisions. The inclusion criteria listed below are based upon the PCC strategy:

- Population: adolescents mean age 10 – 19 years old.(17)
- Context: South Asia. The World Bank limits the South Asia region to Afghanistan, Bangladesh, Bhutan, India, Maldives, Nepal, Pakistan, and Sri Lanka.(18)
- Concept: in this proposed scoping review only undernutrition indicators for adolescents will be used (stunting, thinness or underweight, and micronutrient deficiencies(3)). Adolescent undernutrition will be assessed quantitatively using the following WHO indicators: 1) thinness or underweight (low BMI-for-age); 2) stunting (low height-for-age); and 3) micronutrient deficiencies. All studies will be included regardless of the growth references or cut-off values for micronutrients deficiency followed.
- Adolescent undernutrition in South Asia will also be assessed qualitatively, therefore qualitative studies exploring perspectives, experiences or opinions around adolescent undernutrition will be included.
- Type of studies: quantitative and qualitative studies as well as grey literature, e.g. primary research studies, reviews, government reports and guidelines.

Studies will be excluded if they include:

- Only overnutrition indicators (obesity and overweight).
- Pregnant or breastfeeding adolescents.
- Adolescent athletes.
- Adolescents with long term conditions such as diabetes, tuberculosis or HIV.
- Hospitalised adolescents.
- Intervention studies targeting treatment of a specific illness or condition such as diarrhoea.

Stage 3: study selection

The screening process of this proposed scoping review will comprise two phases. First, titles and abstract will be reviewed by two independent reviewers following a broad inclusion criteria, that is, studies looking at adolescent undernutrition in South Asia. Papers identified by either or both reviewers will be included in the next phase, which is the full-text screening. The same reviewers will screen full-text studies using the eligibility criteria mentioned above. Disagreements will be resolved

For peer review only - http://bmjopen.bmj.com/site/about/guidelines.xhtml

by either discussion or referral to a third reviewer. PRISMA flow diagram (see figure 1) will be presented to reflect the search process.

Figure 1: PRISMA Flow Diagram for scoping review process. Retrieved from Peters M, Godfrey C, McInerney P, Soares C, Khalil H, Parker D (2015) The Joanna Briggs Institute reviewers' manual 2015: methodology for JBI scoping reviews.(16)

Stage 4: charting the data

Key characteristics to be extracted from the included studies will be extracted using a predefined data extraction sheet. At this stage, one reviewer (SEQ) will conduct data extraction consisting of a summary table recording the key information of the selected studies (see Table 2).

Study details
• Author(s) / Organization<br>• Year of publication<br>• Country<br>• Province and city (where the study was published or conducted)<br>• Study design<br>• Aims<br>• Sample size
Data relevant to the PCC term
• Population: target population (age, sex)<br>• Context: country<br>• Concept: outcome quantitative measures (stunting, thinness or underweight, and micronutrient deficiencies). Also, note the growth references or the cut-off values for micronutrients deficiency.<br>• Concept: qualitative outcomes (experiences, opinions and perspectives).<br>• Summary of findings.<br>• Recommendations of the author(s)

Table 2: Key information to be extracted.

Stage 5: collating, summarising and reporting the results

The results of the review will be reported as a map of the data extracted in a tabular form showing descriptive statistics. The tables may display the results as distribution of studies per year, geographical area where the study was conducted, location (community, hospital or school based),

For peer review only - http://bmjopen.bmj.com/site/about/guidelines.xhtml

target population, outcomes, and methods. In addition, a narrative description of both quantitative and qualitative results will accompany the descriptive presentation of the results by grouping the data into meaningful summaries for better exposition of the findings. For the qualitative studies, thematic analysis will be used to analyse the findings if data permits. Data will be exported into Nvivo and an inductive coding approach will be followed.

Patient and Public Involvement

No patient involved.

DISSEMINATION AND ETHICS

This protocol will provide a high quality overview of the broad literature available on the adolescent undernutrition in South Asia. Thus, the proposed scoping review will methodologically map the evidence on this topic to identify research priorities in South Asia. The proposed review will also facilitate the identification of uncovered disparities among adolescents living in South Asia. The potential gaps could help policy makers and healthcare organizations to design new research questions as well as interventions to improve adolescent malnutrition in South Asia.

Since a scoping review involves a methodical integration and presentation of available resources, this study does not require ethics approval.

AUTHOR CONTRIBUTIONS

SEQ conceived the idea, developed the methods and wrote the first draft of the manuscript. PG, LAK and RI supervised the protocol, contributed to methods, and supported the drafting and editing of the manuscript. SJ contributed meaningfully to design the search strategy (appendix 1). All authors revised and approved the final manuscript.

FUNDING STATEMENT

This work was supported by NIHR grant number 16/136/87. The research was commissioned by the National Institute of Health Research using Official Development Assistance (ODA) funding. The views expressed are those of the author(s) and not necessarily those of the NHS, the NIHR or the Department of Health and Social Care.

For peer review only - http://bmjopen.bmj.com/site/about/guidelines.xhtml

COMPLETING INTERESTS

None declared.

REFERENCES

1. Independent Expert Group of the Global Nutrition Report. 2018 Global Nutrition Report. 2018.
2. WHO. Global nutrition targets 2025: policy brief series (WHO/NMH/NHD/14.2). Geneva; 2014.
3. WHO. What is malnutrition? 2016 [Available from: http://www.who.int/features/qa/malnutrition/en/.
4. WHO. Growth reference data for 5-19 years 2007 [Available from: https://www.who.int/growthref/en/.
5. de Onis M, Onyango AW, Borghi E, Siyam A, Nishida C, Siekmann J. Development of a WHO growth reference for school-aged children and adolescents. Bulletin of the World Health Organization. 2007;85(9):660-7.
6. Christian P, Smith ER. Adolescent Undernutrition: Global Burden, Physiology, and Nutritional Risks. Annals of nutrition & metabolism. 2018;72(4):316-28.
7. NCD Risk Factor Collaboration. Worldwide trends in body-mass index, underweight, overweight, and obesity from 1975 to 2016: a pooled analysis of 2416 population-based measurement studies in 128.9 million children, adolescents, and adults. Lancet (London, England). 2017;390(10113):2627-42.
8. FAO, IFAD, UNICEF, WFP, WHO. The State of Food Security and Nutrition in the World 2018. Building climate resilience for food security and nutrition. FAO; 2018.
9. Caleyachetty R, Thomas GN, Kengne AP, Echouffo-Tcheugui JB, Schilsky S, Khodabocus J, et al. The double burden of malnutrition among adolescents: analysis of data from the Global School-Based Student Health and Health Behavior in School-Aged Children surveys in 57 low- and middle-income countries. American Journal of Clinical Nutrition. 2018;108(2):414-24.
10. Mokdad AH, Forouzanfar MH, Daoud F, Mokdad AA, El Bcheraoui C, Moradi-Lakeh M, et al. Global burden of diseases, injuries, and risk factors for young people's health during 1990-2013: a systematic analysis for the Global Burden of Disease Study 2013. Lancet (London, England). 2016;387(10036):2383-401.
11. Akhtar S. Malnutrition in South Asia-A Critical Reappraisal. Critical reviews in food science and nutrition. 2016;56(14):2320-30.
12. Mak KK, Tan SH. Underweight problems in Asian children and adolescents. European journal of pediatrics. 2012;171(5):779-85.
13. Pasricha S-R, Biggs B-A. Undernutrition among children in South and South-East Asia. J Paediatr Child Health. 2010;46(9):497-503.
14. Khan AA, Bano N, Salam A. Child Malnutrition in South Asia: A Comparative Perspective. South Asian Survey. 2007;14(1):129-45.
15. Arksey H, O'Malley L. Scoping studies: towards a methodological framework. International Journal of Social Research Methodology. 2005;8(1):19-32.
16. Peters M, Godfrey C, McInerney P, Soares C, Khalil H, Parker D. The Joanna Briggs Institute reviewers' manual 2015: methodology for JBI scoping reviews. Adelaide, SA Australia: The Joanna Briggs Institute; 2015. 24 p.
17. WHO. Adolescent health and development 2019 [Available from: http://www.searo.who.int/entity/child_adolescent/topics/adolescent_health/en/.

For peer review only - http://bmjopen.bmj.com/site/about/guidelines.xhtml

18. The World Bank. The World Bank in South Asia 2019 [Available from: http://www.worldbank.org/en/region/sar/overview.

For peer review only

For peer review only - http://bmjopen.bmj.com/site/about/guidelines.xhtml

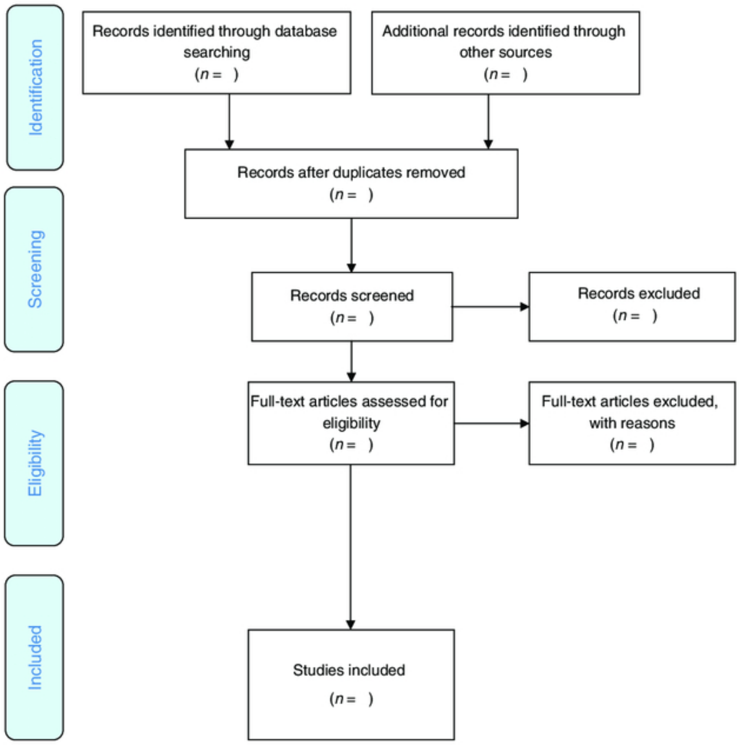

Figure 1: PRISMA Flow Diagram for scoping review process. Retrieved from Peters M, Godfrey C, McInerney P, Soares C, Khalil H, Parker D (2015) The Joanna Briggs Institute reviewers' manual 2015: methodology for JBI scoping reviews.(16)

Appendix 1: search strategy

Medline: ovid

	Searches
1	South Asia*.mp.
2	South East Asia*.mp.
3	exp Afghanistan/
4	exp Bangladesh/
5	exp Bhutan/
6	exp India/
7	Maldives.mp.
8	exp Nepal/
9	exp Pakistan/
10	exp Sri Lanka/
11	1 or 2 or 3 or 4 or 5 or 6 or 7 or 8 or 9 or 10
12	exp Thinness/
13	exp malnutrition/ or exp deficiency diseases/
14	(undernutrition or under nutrition or under-nutrition or stunting or stunted or underweight or under-weight or under weight or thinness or micronutrient deficienc*).mp. [mp=title, abstract, original title, name of substance word, subject heading word, floating sub-heading word, keyword heading word, organism supplementary concept word, protocol supplementary concept word, rare disease supplementary concept word, unique identifier, synonyms]
15	12 or 13 or 14
16	(adolescen* or teen* or youth* or student* or juvenile* or underage or young people or young person or young adult or girl or boy).mp
17	exp Adolescent/
18	16 or 17
19	11 and 15 and 18

Embase: ovid

	Searches
1	exp South Asia/
2	exp Southeastern Asia/
3	exp Afghanistan/
4	exp Bangladesh/
5	exp Bhutan/
6	exp India/
7	exp Maldives/
8	exp Nepal/
9	exp Pakistan/
10	exp Sri Lanka/
11	1 or 2 or 3 or 4 or 5 or 6 or 7 or 8 or 9 or 10
12	exp malnutrition/
13	exp underweight/
14	exp nutritional deficiency/
15	(undernutrition or under nutrition or under-nutrition or stunting or stunted or underweight or under-weight or under weight or thinness or micronutrient deficienc*).mp. [mp=title, abstract, heading word, drug trade name, original title, device manufacturer, drug manufacturer, device trade name, keyword, floating subheading word, candidate term word]
16	12 or 13 or 14 or 15
17	(adolescen* or teen* or youth* or student* or juvenile* or underage or young people or young person or young adult or girl or boy).mp
18	exp adolescence/
19	17 or 18
20	11 and 16 and 19

For peer review only

Cochrane Library

	Searches
1.	South Asia or Afghanistan or Bangladesh or Bhutan or India or Maldives or Nepal or Pakistan or Sri Lanka
2.	malnutrition or undernutrition or under nutrition or under-nutrition or stunting or stunted or underweight or under-weight or under weight or thinness or micronutrient deficienc*
3.	adolescen* or teen* or youth* or student* or juvenile* or underage or young people or young person or young adult or girl or boy
4	#1 AND #2 AND #3

Web of Science

	Searches
1.	TS=(South Asia or Afghanistan or Bangladesh or Bhutan or India or Maldives or Nepal or Pakistan or Sri Lanka)
2.	TS=(malnutrition or undernutrition or under nutrition or under-nutrition or stunting or stunted or underweight or under-weight or under weight or thinness or micronutrient deficienc*)
3.	TS=(adolescen* or teen* or youth* or student* or juvenile* or underage or young people or young person or young adult or girl or boy)
4.	#3 AND #2 AND #1

CINAHL

	Searches
1	“South Asia”
2	(MH “Asia, Western+”)
3	“Afghanistan”
4	“Bangladesh”
5	“Bhutan”
6	“India”
7	“Maldives”
8	“Nepal”
9	“Pakistan”
10	“Sri Lanka”
11	S1 OR S2 OR S3 OR S4 OR S5 OR S6 OR S7 OR S8 OR S9 OR S10
12	(MH "Malnutrition") OR "malnutrition"
13	(MH "Thinness") OR "thinness"
14	"micronutrient deficiency" OR (MH "Vitamin B6 Deficiency") OR (MH "Vitamin B12 Deficiency") OR (MH "Folic Acid Deficiency") OR (MH "Ascorbic Acid Deficiency") OR (MH "Vitamin B Deficiency") OR (MH "Riboflavin Deficiency") OR (MH "Vitamin E Deficiency") OR (MH "Thiamine Deficiency") OR (MH "Vitamin D Deficiency") OR (MH "Vitamin A Deficiency")
15	"undernutrition"
16	"stunting"
17	S11 OR S12 OR S13
18	"adolescents or teenagers or young adults" OR (MH "Young Adult") OR (MH "Adolescence")
	S10 AND S14 AND S15

For peer review only

Psychinfo

	Searches
1	South Asia*.mp.
2	Afghanistan.mp.
3	Bangladesh.mp.
4	Bhutan.mp.
5	India.mp.
6	Maldives.mp.
7	Nepal.mp.
8	Pakistan.mp.
9	Sri Lanka.mp.
10	1 or 2 or 3 or 4 or 5 or 6 or 7 or 8 or 9
11	exp Nutritional Deficiencies/
12	(malnutrition or undernutrition or under nutrition or under-nutrition or stunting or stunted or underweight or under-weight or under weight or thinness or micronutrient deficienc*).mp. [mp=title, abstract, heading word, table of contents, key concepts, original title, tests & measures]
13	11 or 12
14	(adolescen* or teen* or youth* or student* or juvenile* or underage or young people or young person or young adult or girl or boy).mp
15	10 and 13 and 14

Scopus

((TITLE-ABS-KEY (afghanistan) OR TITLE-ABS-KEY (bangladesh) OR TITLE-ABS-KEY (bhutan) OR TITLE-ABS-KEY (india) OR TITLE-ABS-KEY (maldives) OR TITLE-ABS-KEY (nepal) OR TITLE-ABS-KEY (pakistan) OR TITLE-ABS-KEY (sri AND lanka) OR TITLE-ABS-KEY (south AND asia*))) AND ((TITLE-ABS-KEY (youth*) OR TITLE-ABS-KEY (adolescen*) OR TITLE-ABS-KEY (student*) OR TITLE-ABS-KEY (juvenile*) OR TITLE-ABS-KEY (underage) OR TITLE-ABS-KEY (young AND people) OR TITLE-ABS-KEY (young AND person) OR TITLE-ABS-KEY (young AND adult) OR TITLE-ABS-KEY (girl) OR TITLE-ABS-KEY (boy) OR TITLE-ABS-KEY (teen*))) AND ((TITLE-ABS-KEY (malnutrition) OR TITLE-ABS-KEY (undernutrition) OR TITLE-ABS-KEY (under AND nutrition) OR TITLE-ABS-KEY (under-nutrition) OR TITLE-ABS-KEY (stunting) OR TITLE-ABS-KEY (stunted) OR TITLE-ABS-KEY (underweight) OR TITLE-ABS-KEY (under-weight) OR TITLE-ABS-KEY (under AND weight) OR TITLE-ABS-KEY (thinness) OR TITLE-ABS-KEY (micronutrient AND deficienc*))) AND (EXCLUDE (SUBJAREA , "BIOC") OR EXCLUDE (SUBJAREA , "ENVI") OR EXCLUDE (SUBJAREA , "IMMU") OR EXCLUDE (SUBJAREA , "PHAR") OR EXCLUDE (SUBJAREA , "ARTS") OR EXCLUDE (SUBJAREA , "ECON") OR EXCLUDE (SUBJAREA , "PSYC") OR EXCLUDE (SUBJAREA , "ENGI") OR EXCLUDE (SUBJAREA , "NEUR") OR EXCLUDE (SUBJAREA , "DENT") OR EXCLUDE (SUBJAREA , "EART") OR EXCLUDE (SUBJAREA , "CHEM") OR EXCLUDE (SUBJAREA , "ENER") OR EXCLUDE (SUBJAREA , "CENG") OR EXCLUDE (SUBJAREA , "MATH") OR EXCLUDE (SUBJAREA , "VETE") OR EXCLUDE (SUBJAREA , "BUSI") OR EXCLUDE (SUBJAREA , "DECI") OR EXCLUDE (SUBJAREA , "COMP") OR EXCLUDE (SUBJAREA , "MATE") OR EXCLUDE (SUBJAREA , "PHYS") OR EXCLUDE (SUBJAREA , "Undefined"))

For peer review only

Grey literature sources

WHOLIS – the WHO Library Information System	http://kohahq.searo.who.int/
eLENA e-Library of Evidence for Nutrition Actions	https://www.who.int/elena/en/
Opengrey	http://www.opengrey.eu/
WHO	https://www.who.int/en
UNICEF	https://www.unicef.org/
DHS program	https://dhsprogram.com/
Planning and Development Department AJ&K	https://pndajk.gov.pk/
GHDx	http://ghdx.healthdata.org/geography/pakistan
WFP	https://www1.wfp.org/
World Bank eLibrary	https://elibrary.worldbank.org/

For peer review only
